# Synthesis and Characterization of New Counterion-Substituted Triacylgermenolates and Investigation of Selected Metal–Metal Exchange Reactions

**DOI:** 10.1021/acs.organomet.2c00256

**Published:** 2022-07-13

**Authors:** Manfred Drusgala, Matthias Paris, Janine Maier, Roland C. Fischer, Michael Haas

**Affiliations:** Institute of Inorganic Chemistry, Graz University of Technology, Stremayrgasse 9/IV, 8010 Graz, Austria

## Abstract

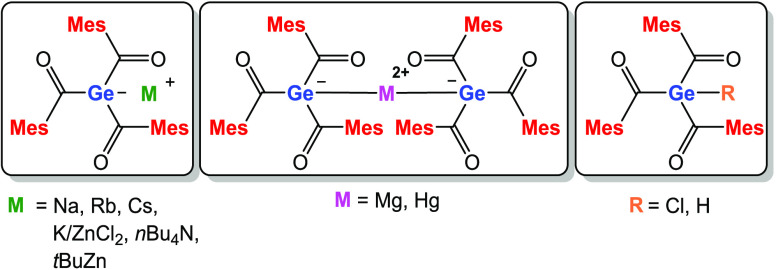

The synthetic process to obtain triacylgermenolates with alternated counterions by single-electron-transfer reactions or by a direct approach is described. The formation of these derivatives was confirmed by NMR spectroscopy and UV–vis spectroscopy. Moreover, metal–metal exchange reactions of potassium-substituted triacylgermenolate **2a** with MgBr_2_, ZnCl_2_, and HgCl_2_ are presented. Additionally, **2a** was reacted with *n*Bu_4_NBr, which led to the formation of ammonia-substituted triacylgermenolate **8**. Furthermore, we reacted **2a** with HCl/Et_2_O to obtain triacylgermane **9**. Subsequently, we investigated the reaction of **9** with *t*Bu_2_Zn and *t*Bu_2_Hg. NMR spectroscopy, single-crystal X-ray crystallography, and UV–vis spectroscopy are employed for analysis of structural properties.

## Introduction

**Scheme 1:**
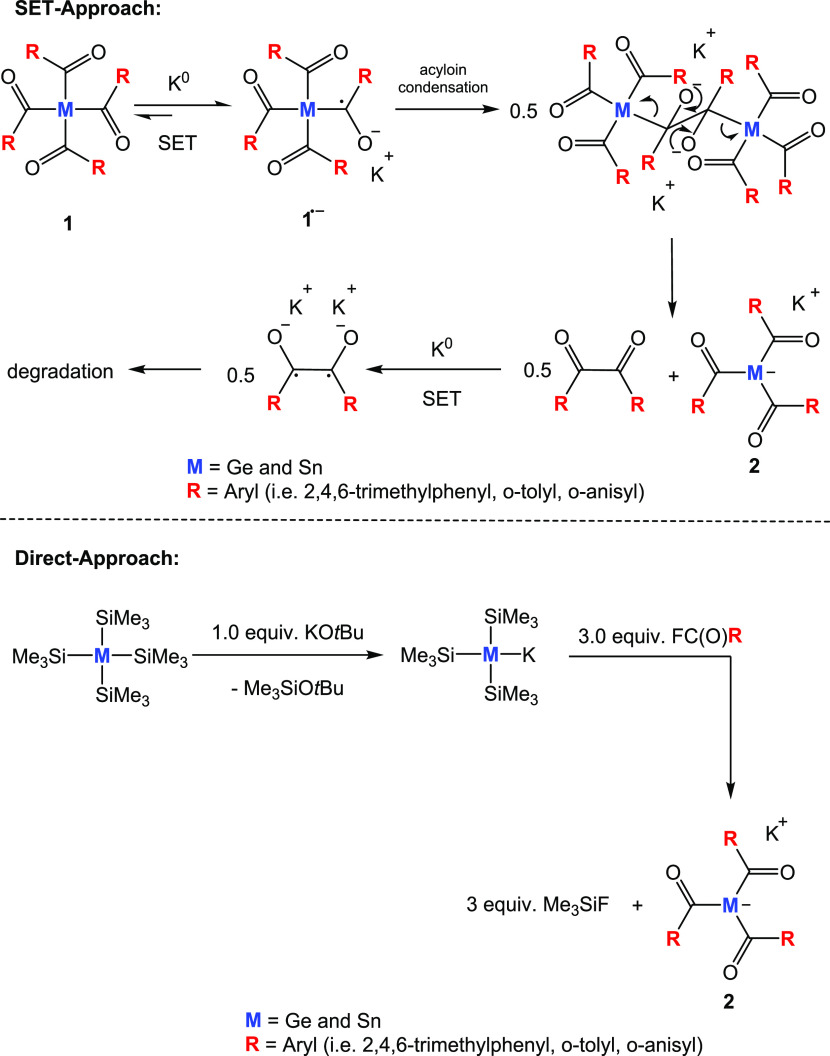
SET Approach vs Direct Approach

Historically speaking, the synthesis and characterization of heavier group 14 (HG 14) enolates were mainly triggered by fundamental investigations in the field of main group chemistry.^1−5^ Recently, we could demonstrate that HG 14 triacylenolates (M = Ge and Sn) represent innovative building blocks for the formation of high-performance free-radical photoinitiators.^6−8^ To synthesize these new HG 14 triacylenolates, we established two pathways. The first methodology uses a potassium-induced single-electron-transfer (SET) approach starting from the respective tetraacyl derivatives. The second method uses the tetrakis(trimethylsilyl) derivatives as starting materials (direct approach). After KO*t*Bu-induced desilylation and a reaction with 3 equiv of an acid fluoride, the respective HG 14 triacylenolates are obtained. The direct approach circumvents the usage of alkali metals and results in higher yields of the target compounds (Scheme 1). In general, the synthesis of HG 14 enolates was focused exclusively on lithium and potassium derivatives.^1−4,9−15^ Other counterions were not investigated so far, although they can significantly influence the reactivity and the structural properties of these enolates. Consequently, the aim of this study was to introduce new counterions. Therein, we focused on germanium as the central atom and used exclusively the 2,4,6-trimethylphenyl moiety as an aromatic group at the carbonyl group.

## Results and Discussion

### Electron-Transfer Reactions of Other Alkali Metals (Method A)

**Scheme 2:**
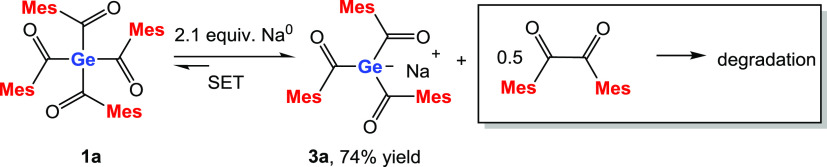
SET Reaction of **1a** with Na^0^

Recently, we have investigated the usage of elemental lithium to induce electron-transfer reactions with tetraacylstannanes. However, only uncharacterizable polymers were found.^16^ The same holds true for tetraacylgermanes. At the beginning of the reaction, the reaction solution changed from yellow to orange, which indicates the formation of the target compound. However, on prolonged stirring (approx. 30 min), the color changed to deep black, along with the formation of a precipitate. We assume that the initially formed lithium-substituted germenolate is highly unstable, and therefore, an isolation of the lithium derivative is not possible. On the basis of this observation, we investigated sodium as a reducing agent. Consequently, we reacted **1a** with 2.1 equiv of sodium in tetrahydrofuran (THF). The reaction solution was stirred overnight, and the complete consumption of the metal marks the end of the reaction. This germenolate **3a** is formed with remarkable selectivity, based on performed NMR spectroscopy at the end of the reaction. **3a** was isolated as a red solid in 74% yield by adding *n*-pentane to the reaction solution (see Scheme 2). Analytic and spectroscopic data that support the structural assignment together with experimental details are summarized in the Experimental Section.

**Scheme 3:**
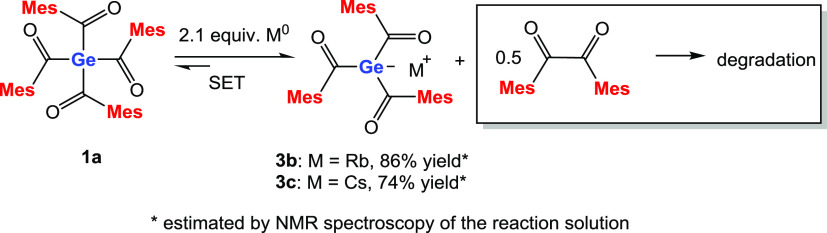
SET Reaction of **1a** with Rb^0^ and Cs^0^

We further investigated the usage of other alkali metals (rubidium, cesium) to synthesize differently decorated triacylgermenolates. In both cases, compound **1a** was reacted with 2.1 equiv of the respective metal in THF as solvent (Scheme 3). However, the high solubility of these heavier alkali metals prevented the isolation of these compounds via the SET approach. Experimental details are summarized in the Experimental Section, and NMR spectra of the reaction solutions are provided in the Supporting Information.

### Direct Approach toward Triacylgermenolates (Method B)

**Scheme 4:**
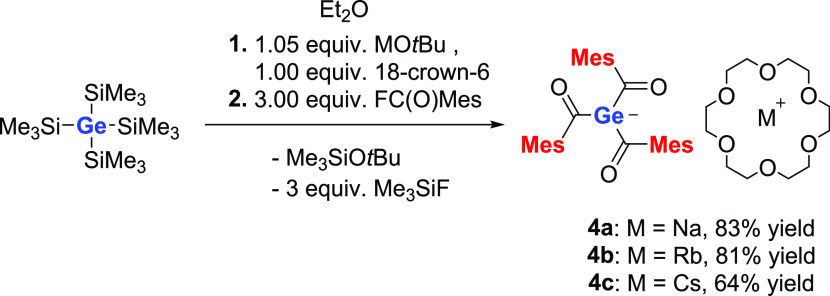
Direct Approach toward **4a–c**

As outlined in the Introduction section, the direct approach is a convenient method to obtain potassium triacylgermenolates, with various substituents on the carbonyl group in good to excellent yields. Here, we used other metal-*tert*-butoxides (NaO*t*Bu, RbO*t*Bu, and CsO*t*Bu) to generate the germanide metal in situ.^17,18^ These germanides were subsequently reacted with 3 equiv of mesitoylfluoride. The addition of 18-crown-6 is necessary to induce precipitation of the formed germenolates in Et_2_O (see Scheme 4). Compounds **4a–c** were isolated in good to excellent yields (experimental details are included in the Experimental Section).

**Scheme 5:**
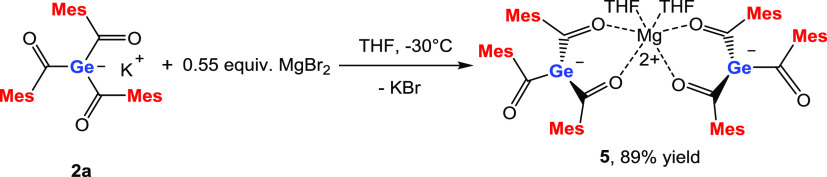
Reaction of **2a** with MgBr_2_

### Stability of **3a** and **4a–c**

All isolated germenolates can be stored in the absence of air at room temperature for prolonged time (usually months).

### UV–vis spectroscopy of **2a**, **3a**, and **4a–c**

**Figure 1:**
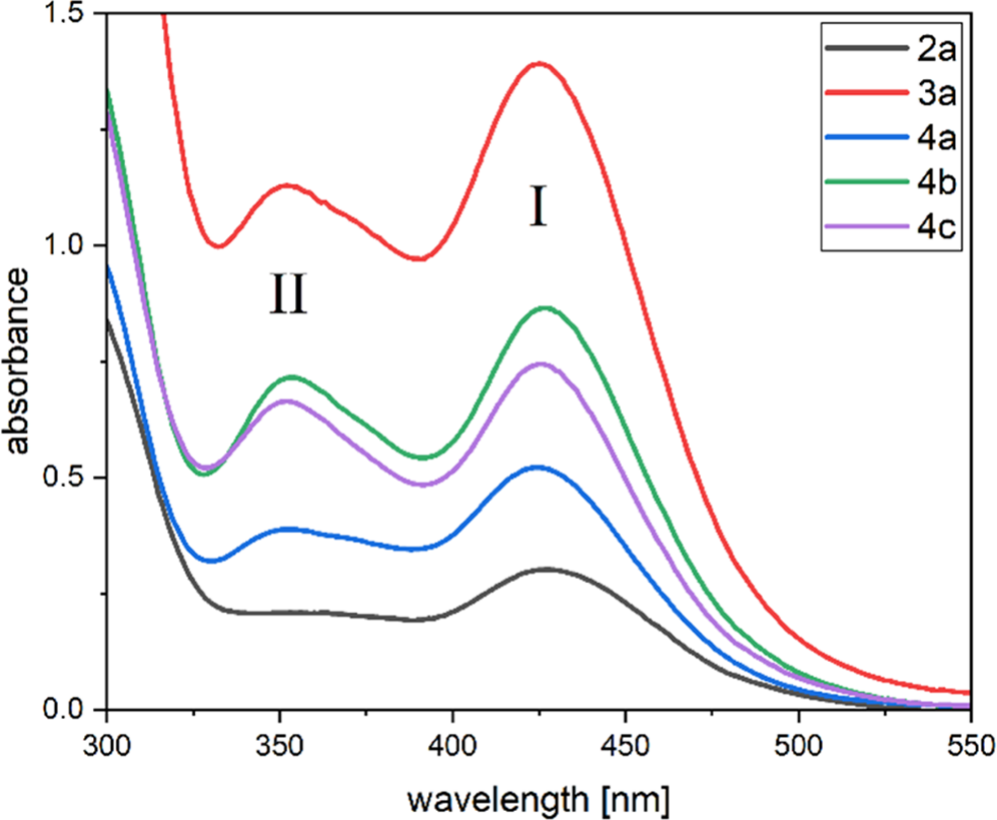
Measured UV–vis spectra of **2a**, **3a**, and **4a**–**c** in THF (1 × 10^–4^ mol/L).

To determine the longest absorption band for our isolated germenolates, we used THF as the solvent and compared it with the parent compound **2a** (M = K). In Figure 1, the measured UV–vis spectra of the isolated germenolates are depicted. These germenolates exhibit two distinct absorption bands with λ_max_ = 425–427 nm (band I [pz-π* excitation]) and 352–353 nm (band II [π-π* excitation]).

### Transmetalation Reactions of **2a**

In contrast to metal enolates where magnesium as a counterion is widely used,^19,20^ an HG 14 magnesium-substituted enolate has not been reported so far. Therefore, we reacted our potassium triacylgermenolate **2a** with 0.55 equiv of MgBr_2_ in THF at −30 °C. After removal of the solvent and resuspension in toluene, the reaction salt was filtered off the reaction solution. Compound **5** was isolated by crystallization in excellent yield (Scheme 5).

**Figure 2:**
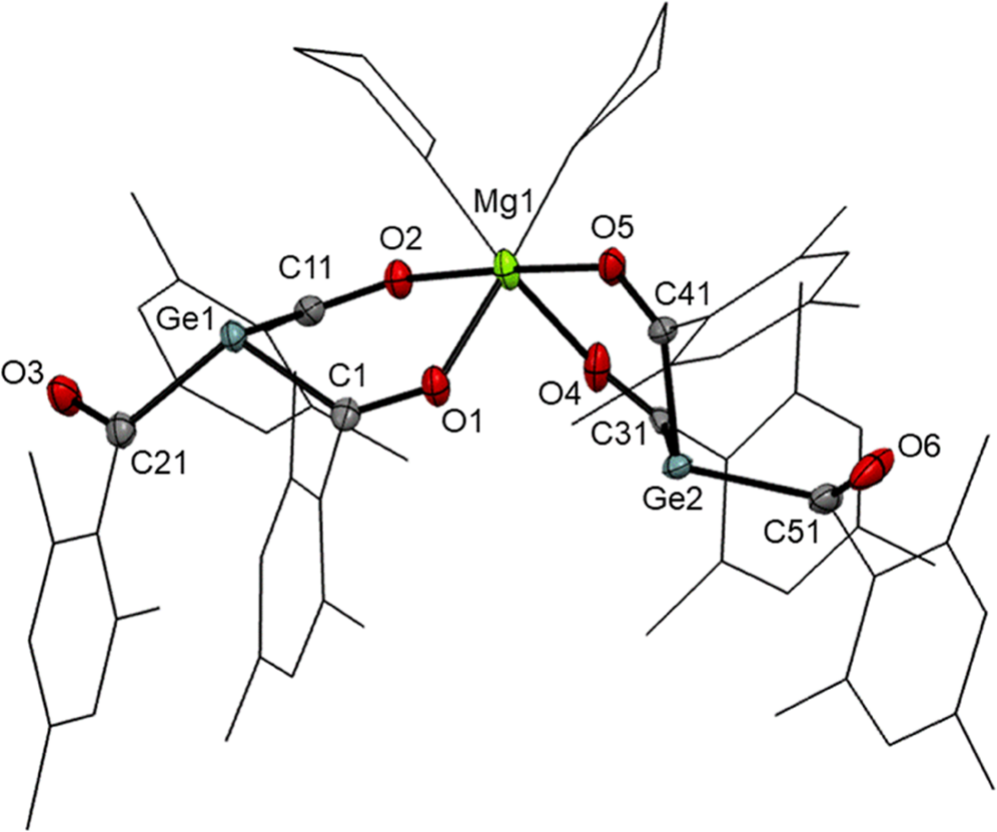
ORTEP representation for compound **5**. Thermal ellipsoids are depicted at the 50% probability level. Hydrogen atoms are omitted, and mesityl groups and THF molecules are displayed as wireframes for clarity. Selected bond lengths (Å) and bond angles (deg) with estimated standard deviations: ∑αGe(1) 308.38, ∑αGe(2) 308.11, Ge(1)–C(1) 2.004 (3), Ge(1)–C(11) 2.006 (3), Ge(1)–C(21) 2.029 (3), C(1)–O(1) 1.252 (3), C(11)–O(2) 1.250 (3), C(21)–O(3) 1.223 (4), Mg(1)–O(1) 2.060 (2), Mg(1)–O(2) 2.043 (2), Mg(1)–O(4) 2.055 (2), Mg(1)–O(5) 2.047 (2), Mg(1)–O(THF) 2.093, C(31)–O(4) 1.167 (9), C(41)–O(5) 1.192 (6), C(51)–O(6) 1.220 (7), Ge(2)–C(31) 2.007 (8), Ge(2)–C(41) 1.988 (5), Ge(2)–C(51) 2.036 (6).

Furthermore, we were able to structurally confirm compound **5** by single-crystal X-ray diffraction analysis (compare Figure 2). **5** crystallizes in monoclinic space group *P*21̅*n* and the unit cell contains four molecules. In close analogy to other structurally characterized germenolates,^6−9,12,13^ the central Ge atoms are pyramidal and have elongated Ge–C single bonds. Noteworthy is an interesting structural feature in the structure of **5**. The relative orientation of the six carbonyl groups is different. While four groups are orientated to the magnesium center, the remaining other two groups do not show any coordination. This coordination is also found in solution, as all signals for the mesityl groups in the ^1^H and ^13^C NMR spectra are in the 2:1 ratio (the solvent for NMR spectra is THF-*d*_8_). Furthermore, two additional donor molecules of THF coordinate to the magnesium atom (experimental details are included in Experimental Section).

**Figure 3:**
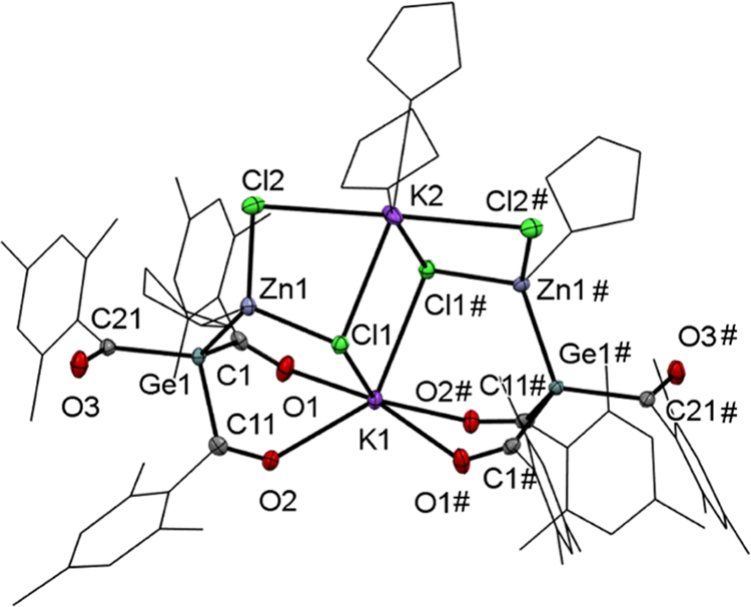
ORTEP representation for compound **6**. Thermal ellipsoids are depicted at the 50% probability level. Hydrogen atoms are omitted, and mesityl groups and THF molecules are displayed as wireframes for clarity. Selected bond lengths (Å) and bond angles (deg) with estimated standard deviations: ∑αGe(1) 317.91, Ge(1)–C(1) 2.050 (3), Ge(1)–C(11) 2.046 (3), Ge(1)–C(21) 2.048 (3), C(1)–O(1) 1.224 (4), C(11)–O(2) 1.217 (4), C(21)–O(3) 1.221 (4), K(1)–O(1) 2.651 (2), K(1)–O(2) 2.742 (2), Zn(1)–Ge(1) 2.4475 (5), Zn(1)–Cl(1) 2.2793 (9), Zn(1)–Cl(2) (2.2688 9), K(1)–Cl(1) 3.1512 (11), K(2)–Cl(1) 3.1350 (11), K(2)–Cl(2) 3.1236 (9).

**Scheme 6:**
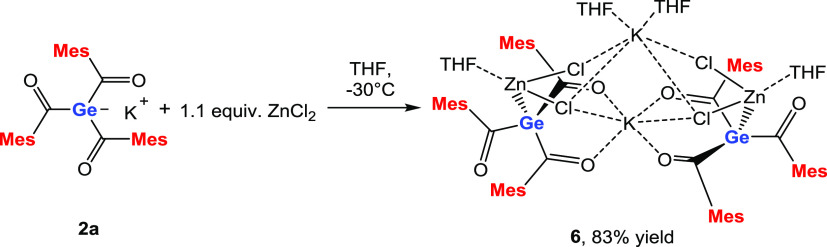
Reaction of **2a** with ZnCl_2_

The next synthetic target was the synthesis of an HG 14 zinc enolate. Therefore, we reacted **2a** with 0.55 equiv of ZnCl_2_. However, NMR spectroscopy performed after the addition of the zinc salt showed the formation of a new product along with the remaining starting material in the ratio of 1:1. Consequently, we added another 0.55 equiv of ZnCl_2_ to the reaction solution and observed the complete consumption of the starting material and the formation of one single product. After removal of the solvent, resuspension in toluene, and filtration, compound **6** was isolated in 83% yield (see Scheme 6). In contrast to the magnesium enolate, compound **6** shows only one signal for the three carbonyl groups and four for the aryl carbon atoms in the ^13^C NMR spectrum. This indicates a better solvent separation for this compound. NMR spectra and detailed assignments are provided in Experimental Section and in the Supporting Information.

Single crystals suitable for X-ray analysis were obtained by cooling the concentrated solution of **6** in THF to −30 °C (Figure 3). Compound **6** crystallized in the monoclinic space group **C*2̅*c**, and the unit cell contains four molecules. Again, the central germanium atoms are pyramidal and the Ge–C bonds are elongated. The structural analysis also sheds light on the experimental observations that no salt was formed. In contrast to the expected transmetalation, the first bimetallic HG 14 enolate is formed. This so-called zincate forms a dimer bridged by two potassium atoms, which have two different coordination modes. K(1) is coordinatively saturated by two chlorine atoms and four oxygen atoms. K(2) is coordinated by two THF molecules and four chlorine atoms. Moreover, the Zn–Ge bond length of 2.448 Å is slightly longer than their covalent radii (2.42 Å) and significantly longer than those of [Ph_3_Ge]_2_Zn,^21^ (Me_3_Si)_3_GeZnCl,^22^ and [(Me_3_Si)_3_Ge]_2_Zn.^23^

### Reaction of **2a** with HgCl_2_

**Figure 4:**
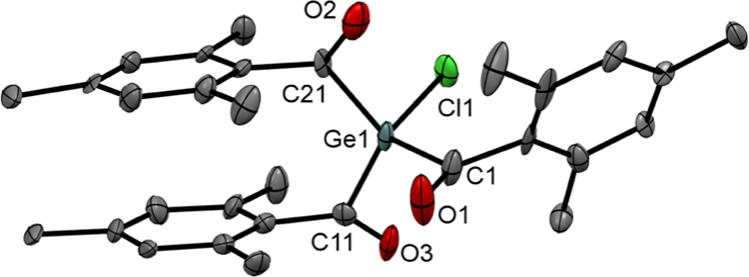
ORTEP representation for compound **7**. Thermal ellipsoids are depicted at the 50% probability level. Hydrogen atoms are omitted for clarity. Selected bond lengths (Å) with estimated standard deviations: Ge(1)–Cl(1) 2.173(3), Ge(1)–C(1) 2.018(11), Ge(1)–C(11) 2.070 (11), Ge(1)–C(21) 2.023(11), C(1)–O(1) 1.215(15), C(11)–O(3) 1.201(14), C(21)–O(2) 1.175(14).

**Scheme 7:**
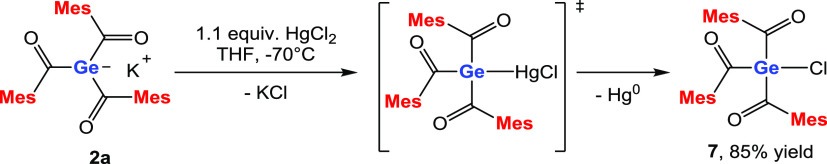
Reaction of **2a** with HgCl_2_

On the basis of the observed reactivity with ZnCl_2_, we wanted to investigate the outcome of the reaction of **2a** with mercury dichloride. Therefore, we reacted **2a** with equimolar amounts of HgCl_2_ at −70 °C in THF (see Scheme 7). After removal of the solvent and the formed salts, the reaction control by NMR spectroscopy showed the formation of a sole germanium-based product with a characteristic shift for an acylgermane (^13^C NMR shifts for the carbonyl group δ = 227.86 ppm). We consequently assumed that the expected Ge–Hg–Cl bond was formed. However, structural analysis revealed our preliminary assumption to be wrong. Instead of the expected product, chloro-trimesitoylgermane **7** was formed in good yields (see Figure 4). Here, we assume that the initial compound is thermally unstable and eliminates elemental mercury. Satgé and co-workers found a similar reactivity for their germanium–mercury derivative.^24^

Compound **7** crystallized in the monoclinic space group *P*21 and the unit cell contains 14 molecules. Additionally, this compound represents an interesting new building block as it can be used as the precursor for further derivatization.

### Reaction of **2a** with Tetrabutylammonium Bromide

**Scheme 8:**
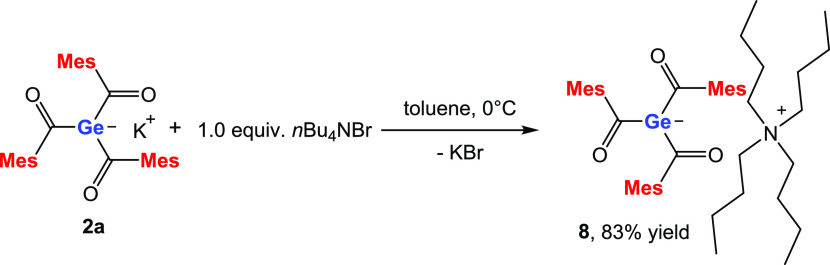
Reaction of **2a** with *n*Bu_4_NBr

In several conferences, we were asked about the reactivity of our germenolates with ammonium salts. As solubility is always an issue for this type of compounds, we thought that the ammonium counterions can contribute to solving this problem. Consequently, we set out and reacted **2a** with equimolar amounts of *n*Bu_4_NBr in toluene at 0 °C. After removal of the salts, compound **8** was isolated in 83% yield as a red oil (see Scheme 8). NMR spectra and detailed assignments are provided in Experimental Section and in the Supporting Information. As expected, compound **8** has good solubility in polar as well as nonpolar solvents. Moreover, compound **8** is highly stable, as no degradation was observed even at room temperature.

### Reaction of **2a** with HCl

**Scheme 9:**
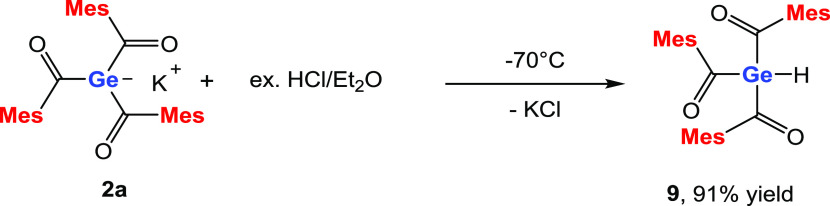
Reaction of **2a** with HCl/Et_2_O

Given the well-known reactivity of germanides with protic solvents to form germanes,^25,26^ we investigated the reaction of **2a** with MeOH, EtOH, and H_2_O. With these above-mentioned reagents, we observed the formation of the expected product; however, we also found the formation of multiple uncharacterized side products. Therefore, we set out and tested the reaction with HCl dissolved in Et_2_O. To our delight, we found more selective reactivity and compound **9** was isolated in excellent yields (see Scheme 9). NMR spectra and detailed assignments are provided in Experimental Section and in the Supporting Information. A characteristic of compound **9** is the significant low-field-shifted ^1^H NMR signal for the hydrogen bonded to the germanium atom with δ = 6.29 ppm.

The so-obtained compound **9** is also an interesting precursor molecule, as the labile Ge–H bond can be used for the formation of selected examples of oligoacyldigermanes. Therefore, we reacted **9** with an organozinc and an organomercury reagent.

### Reaction of **9** with *t*Bu_2_Zn

**Figure 5:**
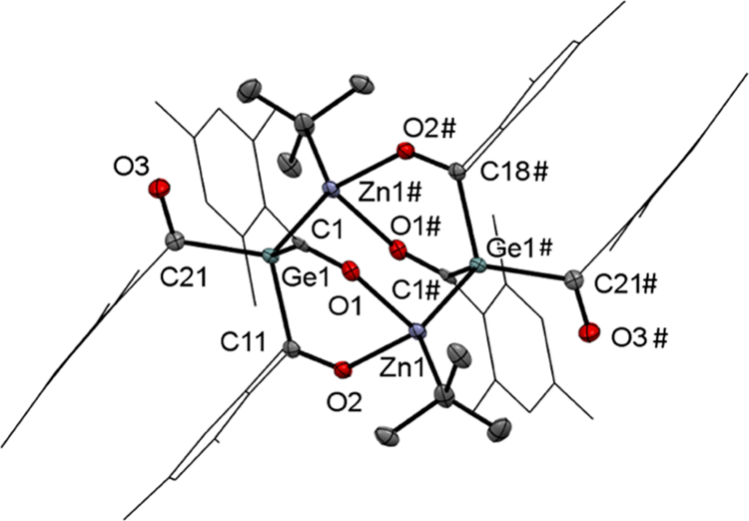
ORTEP representation for compound **10**. Thermal ellipsoids are depicted at the 50% probability level. Hydrogen atoms are omitted, and the mesityl groups are displayed as wireframes for clarity. Selected bond lengths (Å) and bond angles (deg) with estimated standard deviations: ∑αGe(1) 313.89, Ge(1)–C(1) 2.019 (3), Ge(1)–C(11) 2.026 (18), Ge(1)–C(21) 2.050 (19), C(1)–O(1) 1.242 (2), C(11)–O(2) 1.244 (2), C(21)–O(3) 1.215 (2), Zn(1)–O(1) 2.1211 (13), Zn(1)–O(2) 2.1181 (13), Zn(1)–Ge(1) 2.4902 (3).

Following the seminal work of Apeloig and co-workers who presented the first examples of radical activation of the Si–H bond with organozinc reagents,^27^ we reacted **9** with 0.5 equiv of *t*Bu_2_Zn. After the addition of the organozinc reagent, an orange precipitate was immediately formed, which was filtered off. However, in the reaction solution, we found significant amounts of unreacted starting material, and moreover, *t*Bu_2_Zn was completely consumed. Therefore, we attempted to characterize the orange precipitate and found that the compound decomposes immediately in polar solvents (i.e., THF, Et_2_O) to a complex product mixture with no traces of the desired product. In nonpolar solvents, the compound has very low solubility, which prevented complete characterization. However, the ^1^H NMR spectrum indicated that compound **9** reacts with *t*Bu_2_Zn to form an unexpected product. Unfortunately, it was not possible to obtain a ^13^C NMR spectrum of sufficient quality due to low solubility. First, we assumed that this product is an intermediate as a significant amount of starting material was still found in the reaction solution. Consequently, we prolonged the stirring at room temperature (48 h) and changed the reaction conditions (90 °C for 24 h), but no other product was found. The structural determination shed light on the structure of this compound. In Figure 5, the structure of compound **10** is depicted. As assumed, the triacylgermane reacts with *t*Bu_2_Zn, but after the first radical reaction, the zinc atom is coordinatively saturated by two oxygen atoms and this prevented a further reaction of this compound. Moreover, based on the sterical hindrance, we found three signals for the mesityl protons in the ^1^H NMR spectrum.

**Scheme 10:**
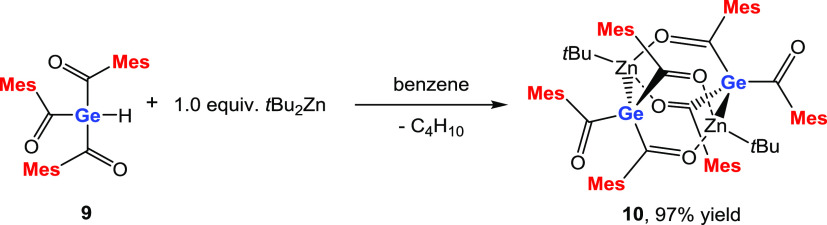
Reaction of **9** with *t*Bu_2_Zn

Compound **10** crystallized in the triclinic space group *P̅*1, and the unit cell contains two molecules. The central germanium atoms are again pyramidal, and the Ge–C bonds are significantly elongated. Moreover, the Zn–Ge bond length is significantly elongated in comparison to their covalent radii and comparable examples.^21−23^ On the basis of the structural analysis, we re-evaluated our reaction and reacted **9** with equimolar amounts of *t*Bu_2_Zn to determine the selectivity of this reaction. To our delight, we found that the reaction is very selective and compound **10** was isolable nearly quantitatively (see Scheme 10).

### Reaction of **9** with *t*Bu_2_Hg

**Scheme 11:**
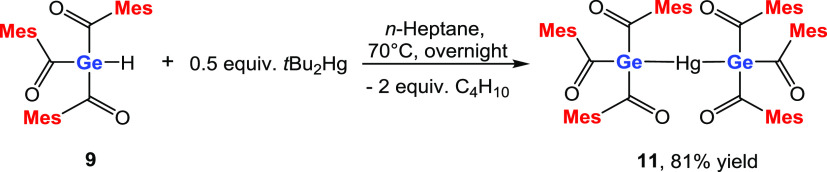
Reaction of **9** with *t*Bu_2_Hg

With *t*Bu_2_Hg, the metalation of the Ge–H bond was much smoother. Compound **9** was reacted in *n*-heptane with *t*Bu_2_Hg and stirred at 70 °C for 18 h. The corresponding digermylmercury compound **11** was obtained in 81% yield (see Scheme 11). NMR spectra and detailed assignments are provided in Experimental Section and in the Supporting Information.

### NMR Spectroscopy

The observed ^13^C NMR shifts of the carbonyl C atoms of all isolated germenolates **2a**, **3a–c**, **4a–c**, **5**, **6**, and **8** were found in the region between δ = 247.83 and 274.88 ppm, which is typical for carbonyl groups attached to the negatively charged germanium atoms. In contrast to this, all carbonyl C atoms of acylgermanes **7**, **9**, and **11** were found significantly high field shifted between δ = 227.86 and 238.17. Again, this correlates well with all carbonyl C shifts of other known acylgermanes (see Table 1).^6,7,28,29^

**Table 1:** ^13^C NMR Shifts of the Carbonyl Atoms for Compounds **3a**–**c**, **4a**–**c**, **5−9**, and **11**

com	^13^C NMR (ppm)	com	^13^C NMR (ppm)	com	^13^C NMR (ppm)
**2a**	263.14^a^	**4b**	261.15^b^	**8**	261.45^b^
**3a**	263.75^a^	**4c**	260.99^b^	**9**	231.20^b^
**3b**	262.13^a^	**5**	247.83^a^	**11**	238.17^b^
			274.88^a^		
**3c**	261.66^a^	**6**	248.75^a^		
**4a**	262.68^b^	**7**	227.86^b^		

a Measured in THF-*d*_8_ at RT.

b Measured in C_6_D_6_ with 18-crown-6 at RT.

## Conclusions

In summary, we investigated the synthesis of a variety of new triacylgermenolates by a single-electron-transfer reaction or by a direct approach. The single-electron-transfer reactions were induced by the respective alkali metals (sodium, rubidium, or cesium). In all cases, the formation of triacylgermenolates (**3a–c**) was observed. However, the high solubility of the rubidium and cesium derivatives prevented the complete isolation. For the direct approach, the respective tris(trimethylsilyl)germanides were synthesized by base-mediated desilylation of tetrakis(trimethylsilyl)germane with metal-*tert*-butoxides (NaO*t*Bu, RbO*t*Bu, and CsO*t*Bu) and reacted with 3 equiv of mesitoylfluoride. The addition of 18-crown-6 was necessary to induce precipitation of the formed germenolate in Et_2_O. Compounds **4a–c** were isolated in good to excellent yields and completely characterized. Furthermore, we performed selected transmetalation of potassium-substituted germenolate **2a** with MgBr_2_, ZnCl_2_, and HgCl_2_. In the case of the magnesium salt, we found the formation of the expected magnesium-bridged derivative **5** in excellent yield, which represents the first magnesium-coordinated HG 14 enolate. ZnCl_2_ reacts with **2a** under the formation of the first bimetallic HG 14 enolate **6**. The attempted transmetalation with HgCl_2_ did not lead to the expected product. Instead, the formation of chloro-trimesitoylgermane **7** was observed. Furthermore, we reacted **2a** with *n*Bu_4_NBr and found selective formation to the corresponding ammonium germenolate **8**. The reaction of **2a** with HCl/Et_2_O led to the formation of the corresponding acylgermane **9**. Compound **9** was reacted with *t*Bu_2_Zn and *t*Bu_2_Hg to synthesize oligoacyldigermanes. While the reaction of **9** with *t*Bu_2_Hg yields the expected digermylmercury compound **11**, the reaction with *t*Bu_2_Zn stopped after the first radical reaction and compound **10** is formed quantitatively. Further studies to investigate the reactivity of these new germenolates are currently in progress. In addition, we are currently testing compound **7** as a new building block for further derivatization.

## Experimental Section

### General Procedures

All experiments were performed under a nitrogen atmosphere using standard Schlenk techniques. Solvents were dried using a column solvent purification system.^30^ Me_3_SiCl (≥99%), GeCl_4_ (>99.99%), KO*t*Bu (>98%), NaO*t*Bu (97%), ClC(O)Mes (98%), 18-crown-6 (99%), potassium (98%), sodium (98%), rubidium (99.6%), cesium (≥99.95%), HCl gas (3.0; 99,9%), THF-*d*_8_ (99.5 atom% D), C_6_D_6_ (99.5 atom%, D), and CDCl_3_ (99.8 atom% D) were used without any further purification. Salts were dried before usage. For the measurement of air-sensitive samples, deuterated solvents were additionally dried (C_6_D_6_ was dried by 24 h reflux above a sodium/potassium alloy; THF-*d*_8_ was dried by 6 h reflux above lithium aluminum hydride). Cesium-*tert*-butoxide,^31^ rubidium-*tert*-butoxide,^31^ di-*tert*-butylmercury^32^ (note: as organomercury compounds are acutely toxic by all exposure routes, all operations involving this compound should be carried out in a certified chemical fume hood or glovebox), di-*tert*-butylzinc,^33^ tetrakis(2,4,6-trimethylbenzoyl)germane,^34^ and potassium-tris(2,4,6-trimethylbenzoyl)germanide•0.5 DME^6^ were prepared according to the published procedures. ^1^H and ^13^C NMR spectra were recorded either on a Varian INOVA 300 MHz, a Bruker AVANCE DPX 200 MHz, or a Bruker Avance 300 MHz spectrometer in C_6_D_6_ or THF-*d*_8_ solution or with D_2_O capillary and referenced vs TMS using the internal 2H-lock signal of the solvent. Infrared spectra were obtained on a Bruker α-P Diamond ATR Spectrometer from the solid sample. Melting points were determined using a Stuart SMP50 apparatus and were uncorrected. Elemental analyses were carried out on a Hanau Vario Elementar EL apparatus. UV–vis absorption spectra were recorded on a PerkinElmer Lambda 5 spectrometer.

### X-ray Crystallography

All crystals suitable for single-crystal X-ray diffractometry were removed from a vial or Schlenk flask and immediately covered with a layer of silicone oil. A single crystal was selected, mounted on a glass rod on a copper pin, and placed in a cold N_2_ stream. XRD data collection for compounds **5**, **6**, **7**, and **10** was performed on a Bruker APEX II diffractometer with the use of an Incoatec microfocus sealed tube of Mo Kα radiation (λ = 0.71073 Å) and a CCD area detector. Empirical absorption corrections were applied using SADABS or TWINABS.^35,36^ The structures were solved with either the use of direct methods or the intrinsic phasing option in SHELXT and refined by the full-matrix least-squares procedures in SHELXL^37−39^ or Olex2.^40^ The space group assignments and structural solutions were evaluated using PLATON.^41,42^ Nonhydrogen atoms were refined anisotropically. Hydrogen atoms were either located in a difference map or in calculated positions corresponding to the standard bond lengths and angles. The disorder was handled by modeling the occupancies of the individual orientations using free variables to refine the respective occupancy of the affected fragments (PART).^43^Table S1 in the Supporting Information contains crystallographic data and details of measurements and refinement for all compounds. Crystallographic data (excluding structure factors) have been deposited with the Cambridge Crystallographic Data Centre (CCDC) under the following numbers (**5**, 2174956; **6**, 2174957; **7**, 2174958; **10**, 2174959)

#### Synthesis of **3a**

To a solution of 500 mg of tetrakis(2,4,6-trimethylbenzoyl)germane (0.76 mmol; 1.00 equiv) in 10 mL of THF, 37 mg of sodium (1.59 mmol; 2.10 equiv) was added at −70 °C. The reaction mixture was brought to room temperature and stirred overnight, turning into a red solution. The next day, the product was precipitated out of solution using *n*-pentane. It was filtrated off, subsequently, giving the product as a red solid. Yield: 300 mg (0.56 mmol; 74%) of analytically pure **3a** as a red crystalline solid. Mp: decomposition > 200 °C. Anal. calcd (%) for C_30_H_33_GeNaO_3_: C, 67.07; H, 6.19, found: C, 67.23; H, 6.21. ^13^C NMR data (THF-*d*_8_, TMS, ppm): 263.75 (*C*=O), 148.43 (Mes-*C4*), 135.52 (Mes-*C1*), 131.75 (Mes-*C2*), 128.63 (Mes-*C3*), 21.42, 20.46 (Aryl-*C*H_3_). ^1^H NMR data (THF-*d*_8_, TMS, ppm): 6.36 (s, 6H, Mes-*H*), 2.11 (s, 9H, Mes-C*H*_3_), 2.00 (s, 18H, Mes-C*H*_3_). UV–vis: λ [nm] (ε [L/mol/cm]) = 425 (13 900), 352 (11 290). IR (neat): ν(C=O) = 1640, 1605.

#### Synthesis of **3b**

To a solution of 100 mg of tetrakis(2,4,6-trimethylbenzoyl)germane (0.15 mmol; 1.00 equiv) in 5 mL of THF, 27 mg of rubidium (0.32 mmol; 2.10 equiv) was added. The reaction mixture was stirred overnight, turning into a red solution. Due to the good solubility of the product, isolation was not possible. Conversion of the starting material was monitored by NMR spectroscopy with D_2_O capillary of the crude reaction solution. Yield: 78 mg (0.13 mmol; 86%) of **3b** estimated by NMR spectroscopy. ^13^C NMR data (D_2_O, TMS, ppm): 262.13 (*C*=O), 147.92 (Mes-*C4*), 134.60 (Mes-*C1*), 131.00 (Mes-*C2*), 127.82 (Mes-*C3*), 20.62, 19.65 (Aryl-*C*H_3_). ^1^H NMR data (D_2_O, TMS, ppm): 6.36 (s, 6H, Mes-*H*), 2.12 (s, 9H, Mes-C*H*_3_), 1.99 (s, 18H, Mes-C*H*_3_).

#### Synthesis of **3c**

To a solution of 100 mg of tetrakis(2,4,6-trimethylbenzoyl)germane (0.15 mmol; 1.00 equiv) in 5 mL of THF, 42 mg of cesium (0.32 mmol; 2.10 equiv) was added. The reaction mixture was stirred overnight, turning into a red solution. Due to the good solubility of the product, isolation was not possible. Conversion of the starting material was monitored by NMR spectroscopy with D_2_O capillary of the crude reaction solution. Yield: 72 mg (0.11 mmol; 74%) of **3c** estimated by NMR spectroscopy. ^13^C NMR data (D_2_O, TMS, ppm): 261.66 (*C*=O), 147.91 (Mes-*C4*), 134.68 (Mes-*C1*), 131.09 (Mes-*C2*), 127.88 (Mes-*C3*), 20.62, 19.65 (Aryl-*C*H_3_). ^1^H NMR data (D_2_O, TMS, ppm): 6.36 (s, 6H, Mes-*H*), 2.11 (s, 9H, Mes-C*H*_3_), 1.99 (s, 18H, Mes-C*H*_3_).

#### Synthesis of **4a**

In total, 1.00 g of tetrakis(trimethylsilyl)germane (2.74 mmol; 1.00 equiv) and 723 mg of 18-crown-6 (2.74 mmol; 1.00 equiv) were dissolved in 25 mL of Et_2_O; then, 276 mg of NaO*t*Bu (2.87 mmol; 1.05 equiv) was added. The solution was stirred at room temperature for 1 h. After full conversion (monitored by NMR spectroscopy), 456 mg of 2,4,6-trimethylbenzoyl fluoride (2.74 mmol, 1.00 equiv) was added and then stirred for 15 min. The second and the third equivalent were added in small portions after another 15 and 30 min, respectively. The reaction is stirred for another 2 h. The orange crystalline product was filtered off, washed with cold Et_2_O, and dried in vacuum. Yield: 1.83 g (2.28 mmol; 83%) of analytically pure **4a** as an orange crystalline solid. Mp: 110–115 °C. Anal. calcd (%) for C_42_H_57_GeNaO_9_: C, 62.94; H, 7.17, found: C, 62.70; H, 7.14. ^13^C NMR data (C_6_D_6_, TMS, ppm): 262.68 (*C*=O), 148.24 (Mes-*C*4), 135.13 (Mes-*C*1), 131.39 (Mes-*C*2), 128.45 (Mes-*C*3), 69.72 ((-*C*H_2_-*C*H_2_-O-)_6_), 21.27, 20.41 (Aryl-*C*H_3_). ^1^H NMR data (C_6_D_6_, TMS, ppm): 6.60 (s, 6H, Mes-*H*), 3.29 (s, 24H, (−C*H*_2_–C*H*_2_–O−)_6_), 2.47 (s, 18H, Mes-C*H*_3_), 2.18 (s, 9H, Mes-C*H*_3_). UV–vis: λ [nm] (ε [L/mol/ cm]) = 425 (5212), 352 (3884). IR (neat): ν(C=O) = 1640, 1605.

#### Synthesis of **4b**

In total, 1.00 g of tetrakis(trimethylsilyl)germane (2.74 mmol; 1.00 equiv) and 723 mg of 18-crown-6 (2.74 mmol; 1.00 equiv) were dissolved in 25 mL of Et_2_O; then, 456 mg of RbO*t*Bu (2.87 mmol; 1.05 equiv) was added. The solution was stirred at room temperature for 1 h. After full conversion (monitored by NMR spectroscopy), 456 mg of 2,4,6-trimethylbenzoyl fluoride (2.74 mmol, 1.00 equiv) was added and then stirred for 15 min. The second and the third equivalent were added in small portions after another 15 and 30 min, respectively. The reaction is stirred for another 2 h. The orange crystalline product was filtered off, washed with cold DME, and dried in vacuum. Yield: 1.91 g (2.21 mmol; 81%) of analytically pure **4b** as an orange crystalline solid. Mp: 71–76 °C. Anal. calcd (%) for C_42_H_57_GeRbO_9_: C, 58.39; H, 6.65, found: C, 58.61; H, 6.62. ^13^C NMR data (C_6_D_6_, TMS, ppm): 261.15 (*C*=O), 148.49 (Mes-*C*4), 134.98 (Mes-*C*1), 131.53 (Mes-*C*2), (Mes-*C*3 superimposed by C_6_D_6_ signals), 70.22 ((−*C*H_2_–*C*H_2_–O−)_6_), 21.28, 20.43 (Aryl-*C*H_3_). ^1^H NMR data (C_6_D_6_, TMS, ppm): 6.61 (s, 6H, Mes-*H*), 3.20 (s, 24H, (−C*H*_2_–C*H*_2_–O−)_6_), 2.50 (s, 18H, Mes-C*H*_3_), 2.19 (s, 9H, Mes-C*H*_3_). UV–vis: λ [nm] (ε [L/mol/cm]) = 427 (8647), 353 (7153). IR (neat): ν(C=O) = 1642, 1606.

#### Synthesis of **4c**

In total, 800 mg of tetrakis(trimethylsilyl)germane (2.19 mmol; 1.00 equiv) and 579 mg of 18-crown-6 (2.19 mmol; 1.00 equiv) were dissolved in 25 mL of Et_2_O; then, 474 mg of CsO*t*Bu (2.30 mmol; 1.05 equiv) was added. The solution was stirred at room temperature for 1 h. After full conversion (monitored by NMR spectroscopy), 364 mg of 2,4,6-trimethylbenzoyl fluoride (2.19 mmol, 1.00 equiv) was added and then stirred for 15 min. The second and the third equivalent were added in small portions after another 15 and 30 min, respectively. The reaction was stirred for another 2 h. The orange crystalline product was filtered off, washed with cold Et_2_O, and dried in vacuum. Yield: 1.27 g (1.39 mmol; 64%) of analytically pure **4c** as an orange crystalline solid. Mp: 72–77 °C. Anal. calcd (%) for C_42_H_57_GeCsO_9_: C, 55.35; H, 6.30, found: C, 55.21; H, 6.28. ^13^C NMR data (C_6_D_6_, TMS, ppm): 260.99 (*C*=O), 148.50 (Mes-*C*4), 134.92 (Mes-*C*1), 131.55 (Mes-*C*2), (Mes-*C*3 superimposed by C_6_D_6_ signals), 70.11 ((−*C*H_2_–*C*H_2_–O−)_6_), 21.30, 20.48 (Aryl-*C*H_3_). ^1^H NMR data (C_6_D_6_, TMS, ppm): 6.60 (s, 6H, Mes-*H*), 3.10 (s, 24H, (−C*H*_2_–C*H*_2_–O−)_6_), 2.51 (s, 18H, Mes-C*H*_3_), 2.18 (s, 9H, Mes-C*H*_3_). UV–vis: λ [nm] (ε [L/mol/cm]) = 425 (7434), 352 (6640). IR (neat): ν(C=O) = 1639, 1607.

#### Synthesis of **5**

To a solution of 400 mg of potassium-tris(2,4,6-trimethylbenzoyl)germanide·0.5 DME (0.67 mmol; 1.00 equiv) in 10 mL of THF, 68 mg of magnesium bromide (0.37 mmol; 0.55 equiv) in 10 mL of THF was added at −30 °C via a syringe. The reaction mixture was brought to room temperature, and the solvent was removed. The crude product was resolved in toluene and filtered via a syringe filter. Then, the toluene was partly removed and the product was recrystallized and isolated. Yield: 313 mg (0.30 mmol, 89%) of analytically pure **5** as a red crystalline solid. Mp: 197–198 °C. Anal. calcd (%) for C_60_H_66_Ge_2_MgO_6_: C, 68.46; H, 6.32, found: C, 68.43; H, 6.63. ^13^C NMR data (THF-*d*_8_, TMS, ppm): 274.88, 247.83 (*C*=O), 146.28, 136.54, 131.82, 128.85. 146.34, 137.11, 132.32, 128.92 (Mes-*C*), 20.82, 21.41, 21.40, 20.13 (Aryl-*C*H_3_). ^1^H NMR data (THF-*d*_8_, TMS, ppm): 6.52 (s, 8H, Mes-*H*), 6.28 (s, 4H, Mes-*H*), 2.19 (s, 12H, Mes-C*H*_3_), 2.12 (s, 6H, Mes-C*H*_3_), 2.08 (s, 24H, Mes-C*H*_3_), 1.87 (s, 12H, Mes-C*H*_3_). UV–vis: λ [nm] (ε [L/mol/cm]) = 435 (17394), 366 (8912). IR (neat): ν(C=O) = 1644, 1606.

#### Synthesis of **6**

To a solution of 400 mg of potassium-tris(2,4,6-trimethylbenzoyl)germanide·0.5 DME (0.67 mmol; 1.00 equiv) in 10 mL of THF, 50 mg of zinc chloride (0.37 mmol; 0.55 equiv) in 10 mL of THF was added at −30 °C via a syringe. The reaction mixture was brought to room temperature, and the conversion was monitored by NMR spectroscopy. Since the starting material was only half-consumed, another 50 mg of zinc chloride (0.37 mmol; 0.55 equiv) in 10 mL of THF was added at −30 °C. After bringing the mixture to room temperature and checking for full consumption of the starting material via NMR spectroscopy, the solvent was removed. The crude product was resuspended in toluene and filtered via a syringe filter. Then, the toluene was removed and the product was isolated. Yield: 380 mg (0.28 mmol; 83%) of analytically pure **6** as a yellow crystalline solid. Mp: decomposition > 170 °C. Anal. calcd (%) for C_60_H_66_Cl_4_Ge_2_K_2_O_6_Zn_2_: C, 52.25; H, 4.82, found: C, 52.52; H, 4.53. ^13^C NMR Data (THF-*d*_8_, TMS, ppm): 248.75 (*C*=O), 145.63 (Mes-*C*4), 137.92 (Mes-*C*1), 133.18 (Mes-*C*2), 129.01 (Mes-*C*3), 21.30, 20.47 (Aryl-*C*H_3_). ^1^H NMR Data (THF-*d*_8_, TMS, ppm): 6.52 (s, 12H, Mes-*H*), 2.17 (s, 18H, Mes-C*H*_3_), 2.04 (s, 36H, Mes-C*H*_3_). UV–vis: λ [nm] (ε [L/mol/cm]) = 401 (2362), 382 (3017). IR (neat): ν(C=O) = 1640, 1609.

#### Synthesis of **7**

To a solution of 500 mg of potassium-tris(2,4,6-trimethylbenzoyl)germanide·0.5 DME (0.83 mmol; 1.00 equiv) in 5 mL of THF, 216 mg of HgCl_2_ (0.92 mmol; 1.1 equiv) was added at −70 °C. The reaction mixture was stirred for about an hour while warming up to room temperature. Subsequently, the solvent was removed in vacuum, and the product was resolved with toluene and filtrated using a syringe filter. Again, the solvent was removed in vacuum and the product was recrystallized using *n*-pentane. After storing the product at −30 °C overnight, **7** was isolated as a yellow solid. Yield: 392 mg (0.71 mmol; 85%) of analytically pure **7** as a yellow crystalline solid. Mp: 115–120 °C. Anal. Calcd (%) for C_30_H_33_ClGeO_3_: C, 65.55; H, 6.05, found: C, 65.52; H, 6.05. ^13^C NMR data (C_6_D_6_, TMS, ppm): 227.86 (*C*=O), 140.29 (Mes-*C*4), 140.16 (Mes-*C*1), 133.73 (Mes-*C*2), 129.29 (Mes-*C*3), 21.08, 19.46 (Aryl-*C*H_3_). ^1^H NMR data (C_6_D_6_, TMS, ppm): 6.47 (s, 6H, Mes-*H*), 2.21 (s, 18H, Mes-C*H*_3_), 1.96 (s, 9H, Mes-C*H*_3_). UV–vis: λ [nm] (ε [L/mol/cm]) = 401 (4262), 380 (6056). **IR** (neat): ν(C=O) = 1653, 1646, 1607.

#### Synthesis of **8**

To a solution of 1.00 g of potassium-tris(2,4,6-trimethylbenzoyl)germanide·0.5 DME (1.67 mmol; 1,00 equiv) in 10 mL of THF, 538 mg of tetrabutylammonium bromide (1.67 mmol; 1.00 equiv) in 10 mL of toluene was added at 0 °C. The mixture was stirred for 30 min at room temperature. Subsequently, the solvent was removed in vacuum, and the product was resuspended in toluene and filtrated using a syringe filter. Then, *n*-pentane was added, resulting in the settling of red oil at the bottom of the flask. The solvents were carefully removed with a syringe, and the remaining oil was dried in vacuum. Yield: 1.05 g (1.39 mmol; 83%) of analytically pure **8** as a red oil. Anal. calcd (%) for C_46_H_69_GeNO_3_: C, 73.02; H, 9.19; N 1.85, found: C, 72.87; H, 9.17; N 1.85. ^13^C NMR data (C_6_D_6_, TMS, ppm): 261.45 (*C*=O), 149.24 (Mes-*C*4), 134.43 (Mes-*C*1), 131.50 (Mes-*C*2), 128.26 (Mes-*C*3), 58.20 (N–CH_2_−), 24.07 (−CH_2_–*C*H_2_–CH_2_−) 21.30 (Aryl-*C*H_3_), 20.53 (−CH_2_–*C*H_2_–CH_3_), 19.97 (Aryl-*C*H_3_), 13.92 (−CH_2_–*C*H_3_). ^1^H NMR data (C_6_D_6_, TMS, ppm): 6.53 (s, 6H, Mes-*H*), 3.08–3.03 (m, 8H, N–C*H*_2_−), 2.43 (s, 18H, Mes-C*H*_3_), 2.16 (s, 9H, Mes-C*H*_3_), 1.35–1.24 (m, 8H, −C*H*_2_–C*H*_2_–C*H*_2_−), 1.21–1.12 (m, 8H, −C*H*_2_–C*H*_2_–C*H*_3_), 0.81 (t, 12H, −C*H*_2_–C*H*_3_). UV–vis: λ [nm] (ε [L/mol/cm]) = 425 (4555), 353 (4569). IR (neat): ν(C=O) = 1648, 1599.

#### Synthesis of **9**

In total, 500 mg of potassium-tris(2,4,6-trimethylbenzoyl)germanide·0.5 DME (0.83 mmol; 1.00 equiv) in 5 mL of THF was added to 5 mL of HCl dissolved in Et_2_O (16.80 mmol; 20.14 equiv, 3.36 M) at −70 °C via a syringe. The reaction mixture was brought to room temperature, and the solvent was removed. The crude product was resolved in toluene and filtered using a syringe filter. After removal of the solvent, the yellow oil was again dissolved in *n*-pentane. The product was then recrystallized at −70 °C and isolated. Yield: 391 mg (0.76 mmol; 91%) of analytically pure **9** as a yellow crystalline solid. Mp: 61–62 °C. Anal. calcd (%) for C_30_H_34_GeO_3_: C, 69.94; H, 6.65, found: C, 69.89; H, 6.62. ^13^C NMR data (C_6_D_6_, TMS, ppm): 231.20 (*C*=O), 142.78 (Mes-*C*4), 139.61 (Mes-*C*1), 133.16 (Mes-*C*2), 129.34 (Mes-*C*3), 21.06, 19.33 (Aryl-*C*H_3_). ^1^H NMR data (C_6_D_6_, TMS, ppm): 6.48 (s, 6H, Mes-*H*), 6.29 (s, 1H, Ge-*H*), 2.17 (s, 18H, Mes-C*H*_3_), 1.99 (s, 9H, Mes-C*H*_3_). UV–vis: λ [nm] (ε [L mol^–1^ cm^–1^]) = 403 (964), 381 (1262). IR (neat): ν(C=O) = 1643, 1606.

#### Synthesis of **10**

To a solution of 300 mg of **9** (0.58 mmol; 1.00 equiv) in 5 mL of benzene, 104 mg of di-*tert*-butylzinc (0.58 mmol; 1.00 equiv), dissolved in 4 mL of benzene, was added. The reaction mixture was stirred for 2 h. The product was filtered off and washed with benzene. Yield: 360 mg (0.28 mmol; 97%) of analytically pure **10** as an orange crystalline solid. Mp: 235–236 °C. Anal. calcd (%) for C_68_H_84_Ge_2_O_6_Zn_2_: C, 64.14; H, 6.65, found: C, 64.37; H, 6.64. ^1^H NMR data (C_6_D_6_, TMS, ppm): 6.43 (s, 4H, Mes-*H*), 6.37 (s, 4H, Mes-*H*), 6.20 (s, 4H, Mes-*H*), 2.58 (s, 12H, Mes-C*H*_3_), 2.21 (s, 12H, Mes-C*H*_3_), 1.97 (s, 12H, Mes-C*H*_3_), 1.95 (s, 18H, Mes-C*H*_3_), 1.75 (s, 18H, C-(C*H*_3_)_3_). IR (neat): ν(C=O) = 1627, 1607.

#### Synthesis of **11**

To a solution of 300 mg of **9** (0.58 mmol; 1.00 equiv) in 10 mL of *n*-heptane, 91 mg of di-*tert*-butylmercury (0.29 mmol; 0.50 equiv) was added. The reaction mixture was brought to 70 °C and stirred overnight. The next day, the reaction mixture was brought to room temperature. The crude product was recrystallized from the reaction solution at −30 °C and filtered off. Yield: 290 mg (0.24 mmol; 81%) of analytically pure **11** as a yellow crystalline solid. Mp: 180–182 °C. Anal. calcd (%) for C_60_H_66_Ge_2_HgO_6_: C, 58.64; H, 5.41, found: C, 58.87; H, 5.43. ^13^C NMR data (C_6_D_6_, TMS, ppm): 238.17 (*C*=O), 144.82 (Mes-*C*4), 139.25 (Mes-*C*1), 132.14 (Mes-*C*2), 129.45 (Mes-*C*3), 21.14, 19.52 (Aryl-*C*H_3_). ^1^H NMR data (C_6_D_6_, TMS, ppm): 6.53 (s, 12H, Mes-*H*), 2.19 (s, 36H, Mes-C*H*_3_), 2.08 (s, 18H, Mes-C*H*_3_). UV–vis: λ [nm] (ε [L mol^–1^ cm^–1^]) = 405 (5094), 389 (6685), 340 (9693). IR (neat): ν(C=O) = 1675, 1645, 1638, 1606.

## Supporting Information Available

NMR spectra and X-ray crystallographic information (PDF)
