# Supplementary material for: Synthesis and Characterization of New Counterion-Substituted Triacylgermenolates and Investigation of Selected Metal–Metal Exchange Reactions
Source: Organometallics. 2022 Jul 13;41(15):2170–9. doi: 10.1021/acs.organomet.2c00256 (PMC9488839; doi:10.1021/acs.organomet.2c00256)
Supplement: Supplementary file 1 — om2c00256_si_001.pdf [file om2c00256_si_001.pdf]

Supporting Information

Synthesis and Characterization of New Counterion Substituted Triacylgermenolates and Investigation of Selected Metal-Metal Exchange Reactions

Manfred Drusgala, Matthias Paris, Janine Maier, Roland C. Fischer and Michael Haas*

Institute of Inorganic Chemistry, Graz University of Technology, Stremayrgasse 9/IV, 8010 Graz (Austria)
*E-mail: michael.haas@tugraz.at

Table of Content

Analytical Section 3

NMR-Spectroscopy 3

Figure S1: ^{1}H- and ^{13}C-NMR spectra of **3a** (THF-d_8 solution, vs ext. TMS, ppm)........ 3

Figure S2: ^{1}H- and ^{13}C-NMR spectra of **3b** (THF solution with D_2O capillary, vs ext. TMS, ppm) 4

Figure S3: ^{1}H- and ^{13}C-NMR spectra of **3c** (THF solution with D_2O capillary, vs ext. TMS, ppm) 5

Figure S4: ^{1}H- and ^{13}C-NMR spectra of **4a** (C_6D_6 solution, vs ext. TMS, ppm)........ 6

Figure S5: ^{1}H- and ^{13}C-NMR spectra of **4b** (C_6D_6 solution, vs ext. TMS, ppm) 7

Figure S6: ^{1}H- and ^{13}C-NMR spectra of **4c** (C_6D_6 solution, vs ext. TMS, ppm)........ 8

Figure S7: ^{1}H- and ^{13}C-NMR spectra of **5** (THF-d_8 solution, vs ext. TMS, ppm) 9

Figure S8: ^{1}H- and ^{13}C-NMR spectra of **6** (THF-d_8 solution, vs ext. TMS, ppm) 10

Figure S9: ^{1}H- and ^{13}C-NMR spectra of **7** (C_6D_6 solution, vs ext. TMS, ppm)........ 11

Figure S10: ^{1}H- and ^{13}C-NMR spectra of **8** (C_6D_6 solution, vs ext. TMS, ppm)........ 12

Figure S11: ^{1}H- and ^{13}C-NMR spectra of **9** (C_6D_6 solution, vs ext. TMS, ppm)........ 13

Figure S12: ^{1}H-NMR spectra of **10** (C_6D_6 solution, vs ext. TMS, ppm)........ 14

Figure S13: ^{1}H- and ^{13}C-NMR spectra of **11** (C_6D_6 solution, vs ext. TMS, ppm)........ 15

UV-vis Spectroscopy........ 16

Figure S14: UV-vis Spectrum of **5** (c = 5 x 10^{-5} M) measured in THF........ 16

Figure S15: UV-vis Spectrum of **6** (c = 5 x 10^{-4} M) measured in THF........ 16

Figure S16: UV-vis Spectrum of **7** (c = 5 x 10^{-4} M) measured in THF........ 17

Figure S17: UV-vis Spectrum of **8** (c = 1 x 10^{-4} M) measured in THF........ 17

Figure S18: UV-vis Spectrum of **9** (c = 5 x 10^{-4} M) measured in toluene........ 18

Figure S19: UV-vis Spectrum of **11** (c = 1 x 10^{-4} M) measured in *n*-hexane........ 18

X-ray Crystallography........ 19

Table S1. Crystallographic data and details of measurements for compounds **5**, **6**, **7** and **10**..... 19

Analytical Section

NMR-Spectroscopy

Figure S1: ^{1}H- and ^{13}C-NMR spectra of **3a** (THF-d_8 solution, vs ext. TMS, ppm)

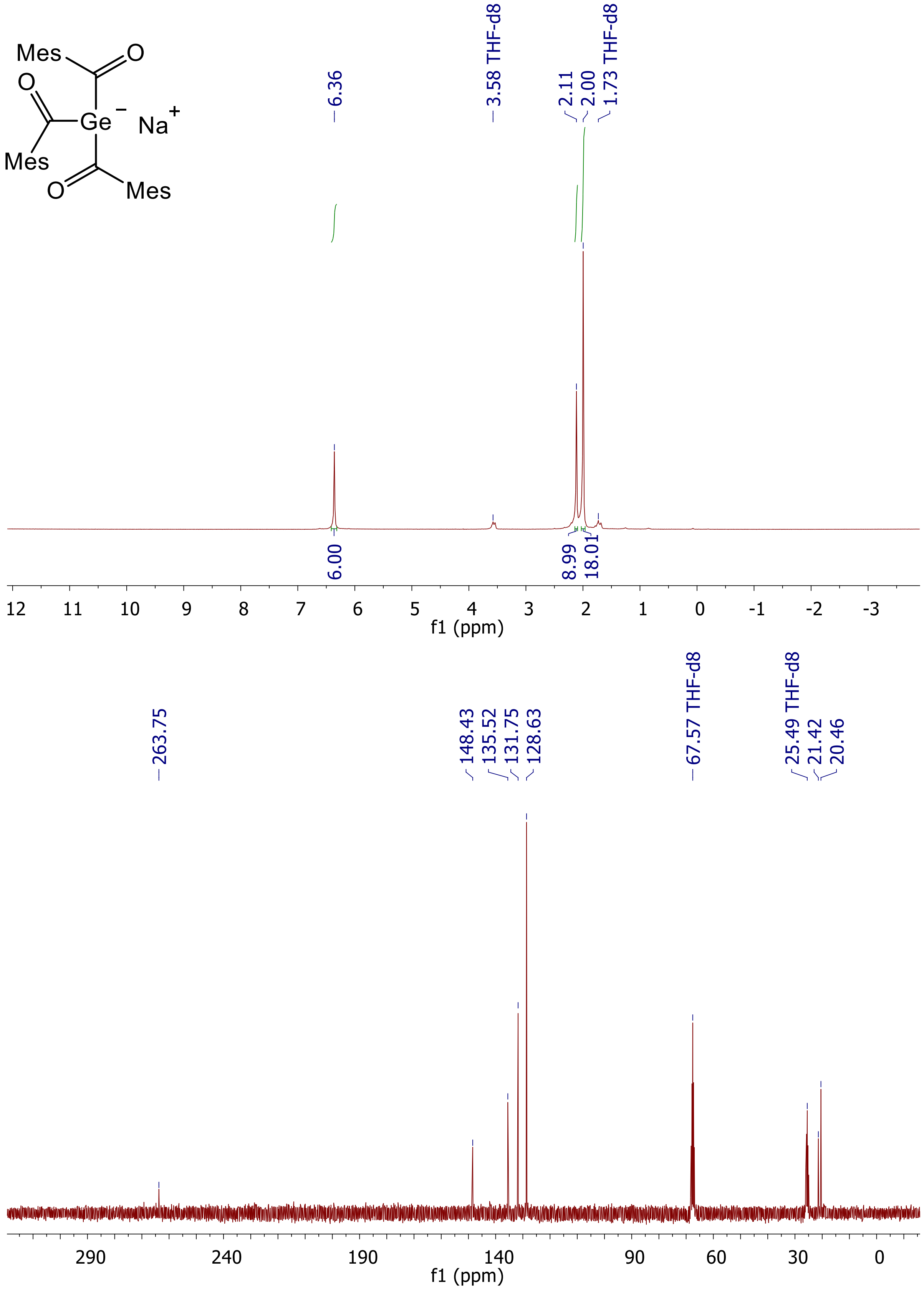

Figure S2: ^{1}H- and ^{13}C-NMR spectra of **3b** (THF solution with D_2O capillary, vs ext. TMS, ppm)

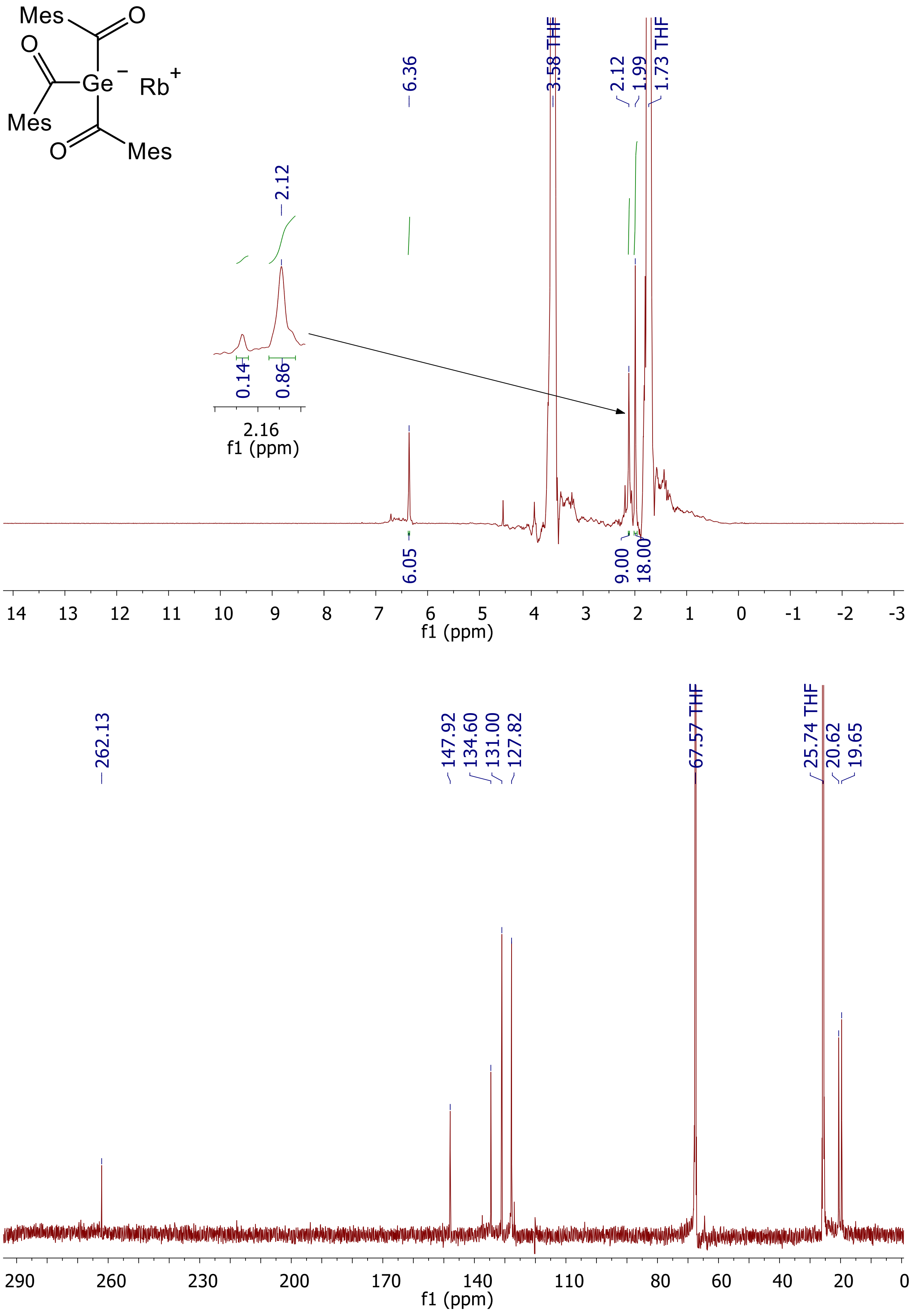

Figure S3: ^{1}H- and ^{13}C-NMR spectra of **3c** (THF solution with D_2O capillary, vs ext. TMS, ppm)

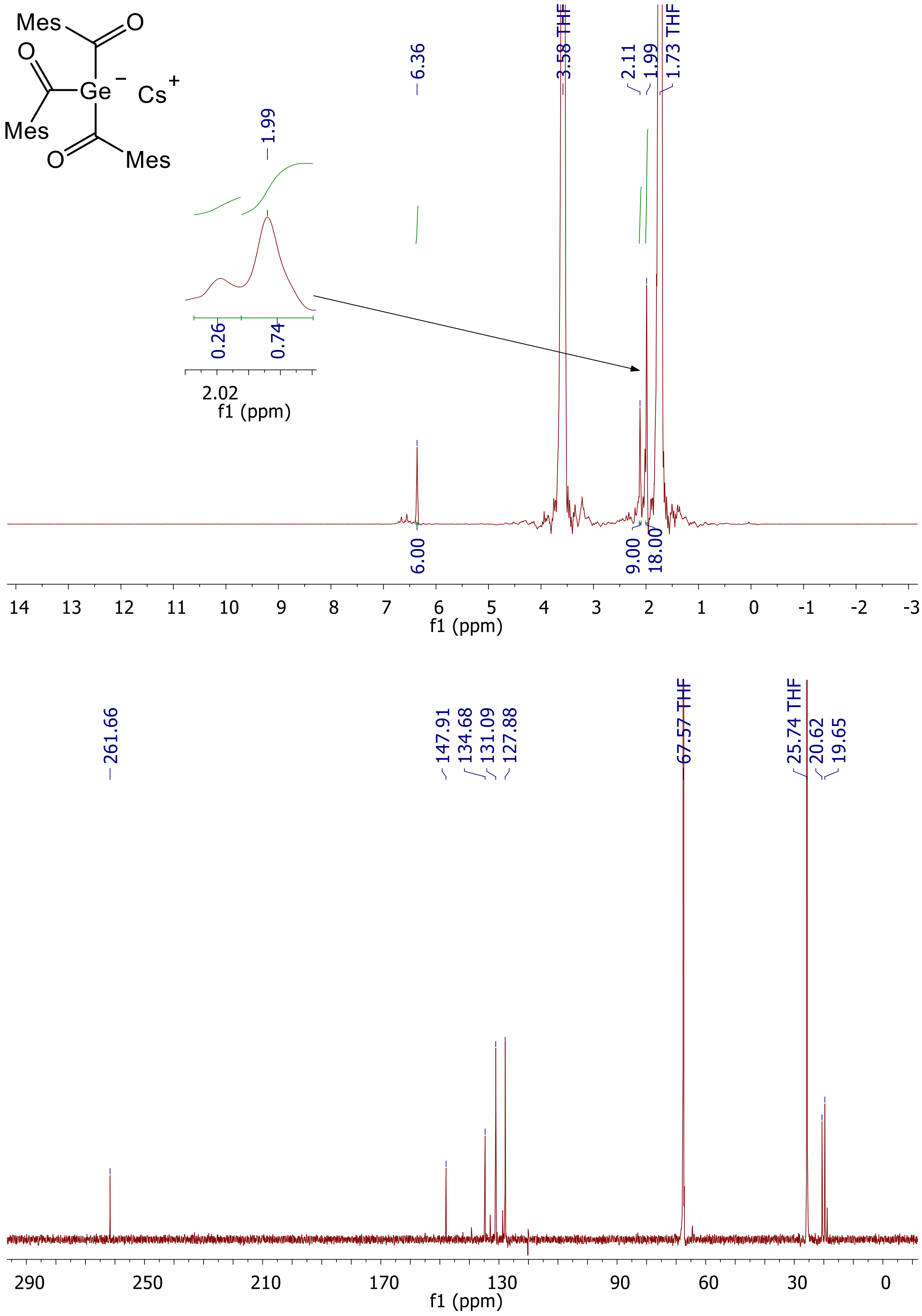

Figure S4: ^{1}H- and ^{13}C-NMR spectra of **4a** (C_6D_6 solution, vs ext. TMS, ppm)

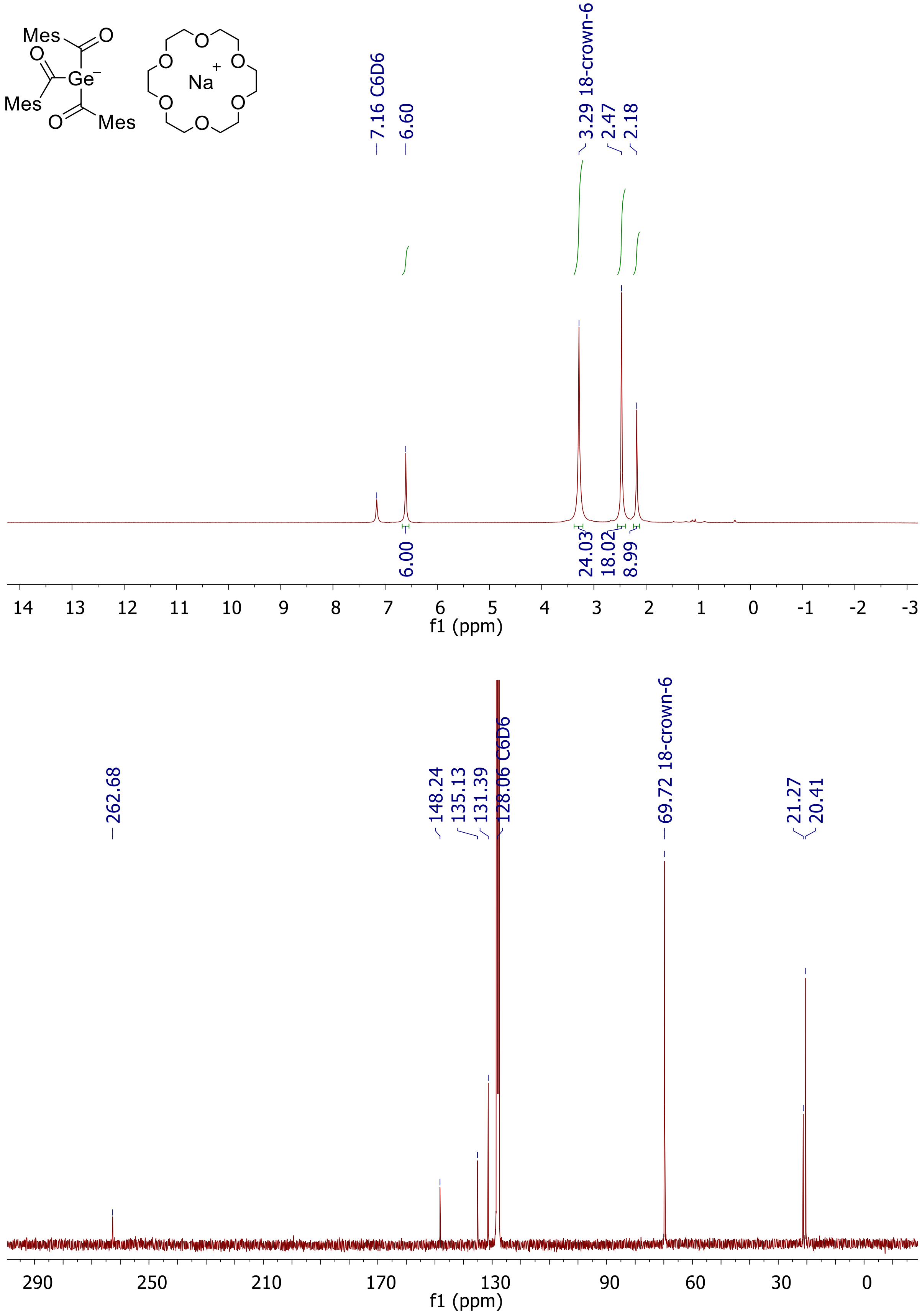

Figure S5: ^{1}H- and ^{13}C-NMR spectra of **4b** (C_6D_6 solution, vs ext. TMS, ppm)

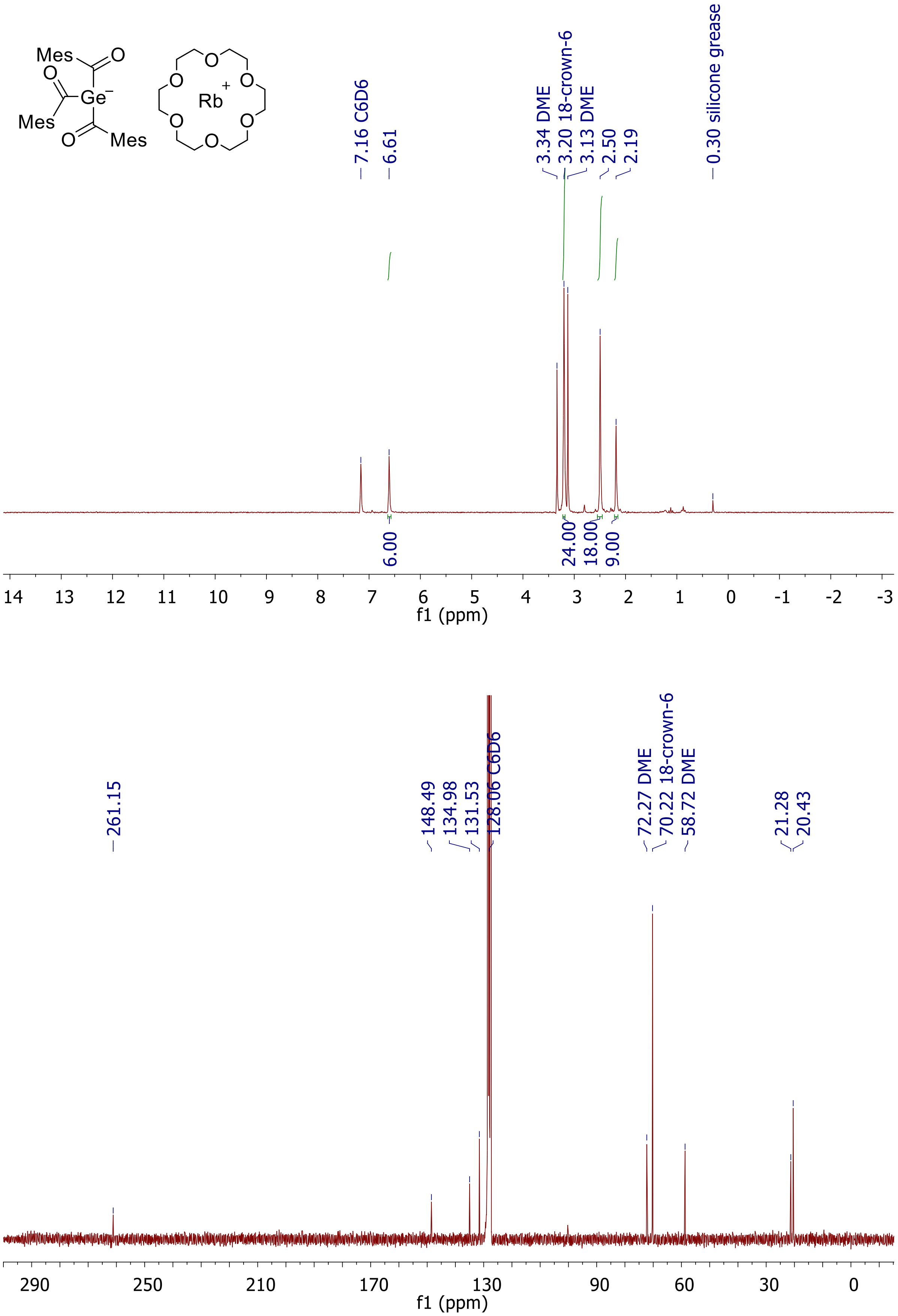

Figure S6: ^{1}H- and ^{13}C-NMR spectra of **4c** (C_6D_6 solution, vs ext. TMS, ppm)

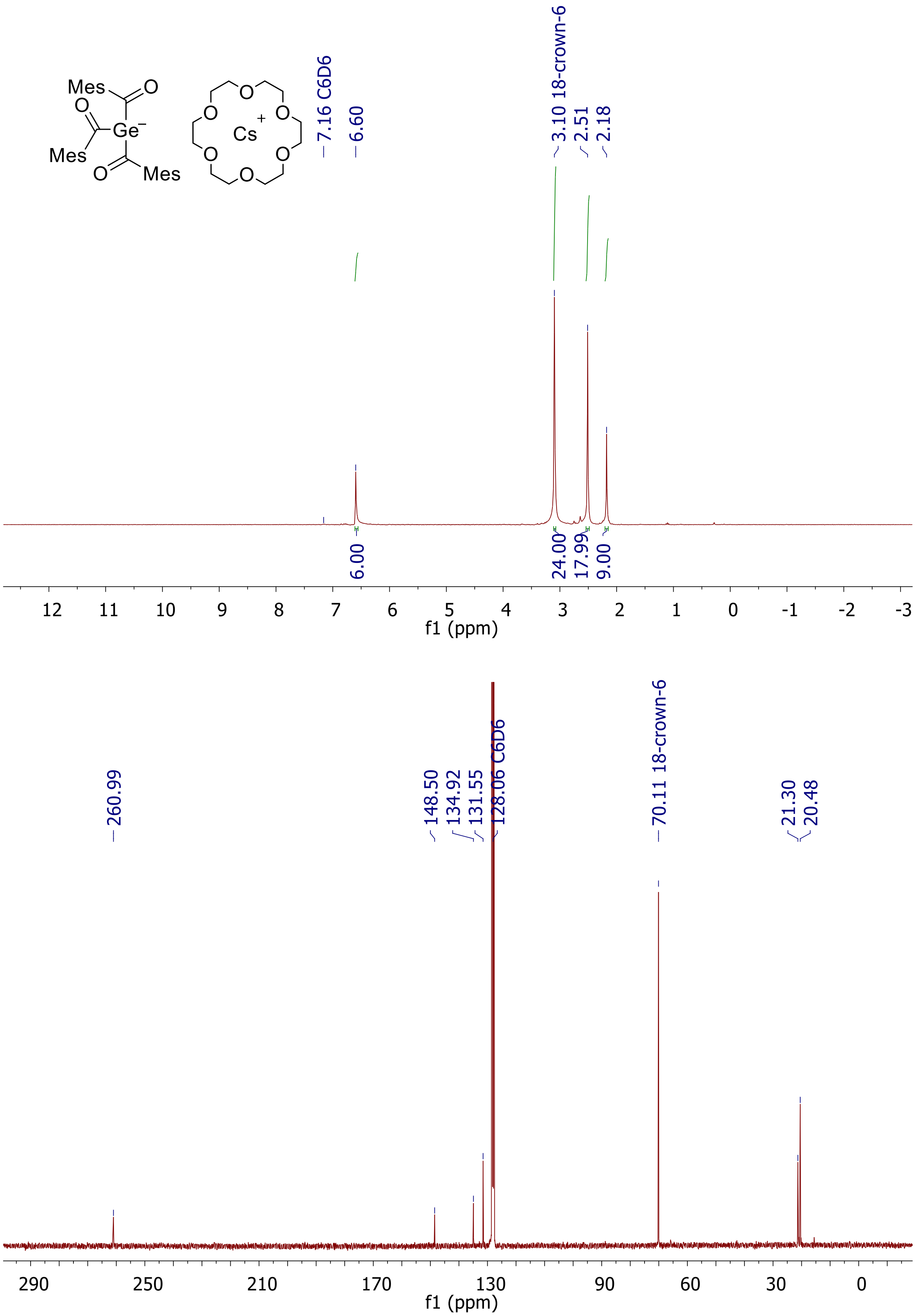

Figure S7: ^{1}H- and ^{13}C-NMR spectra of **5** (THF-d_8 solution, vs ext. TMS, ppm)

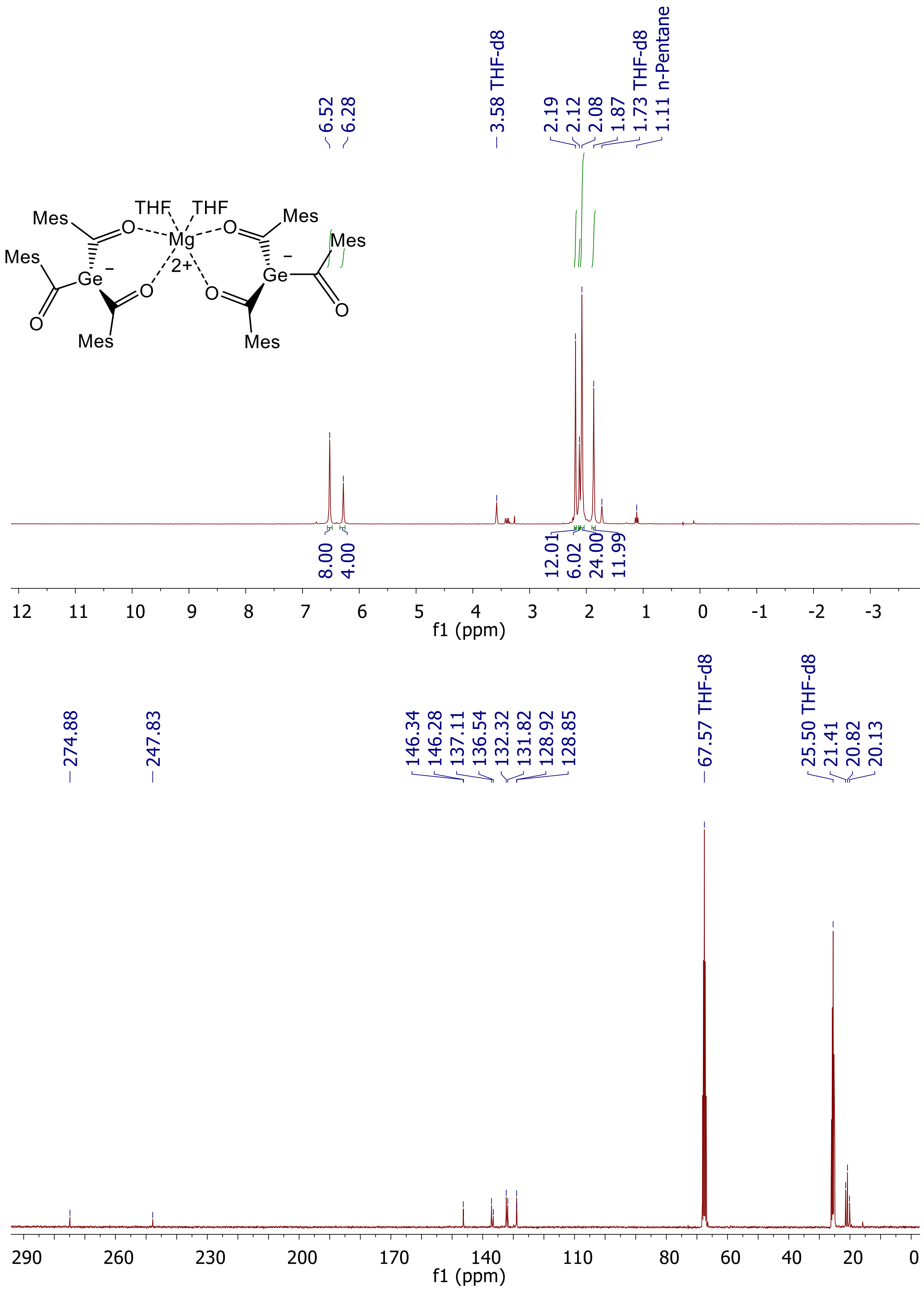

Figure S8: ^{1}H- and ^{13}C-NMR spectra of **6** (THF-d_8 solution, vs ext. TMS, ppm)

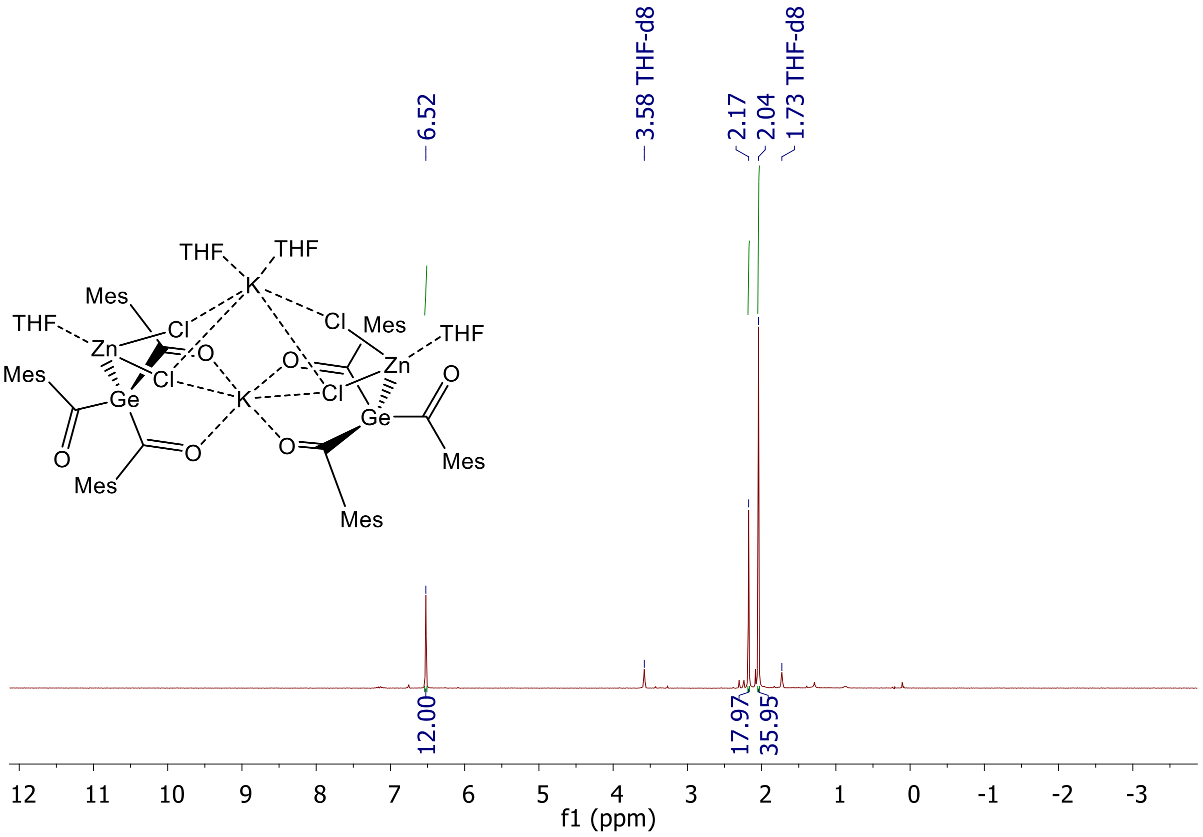

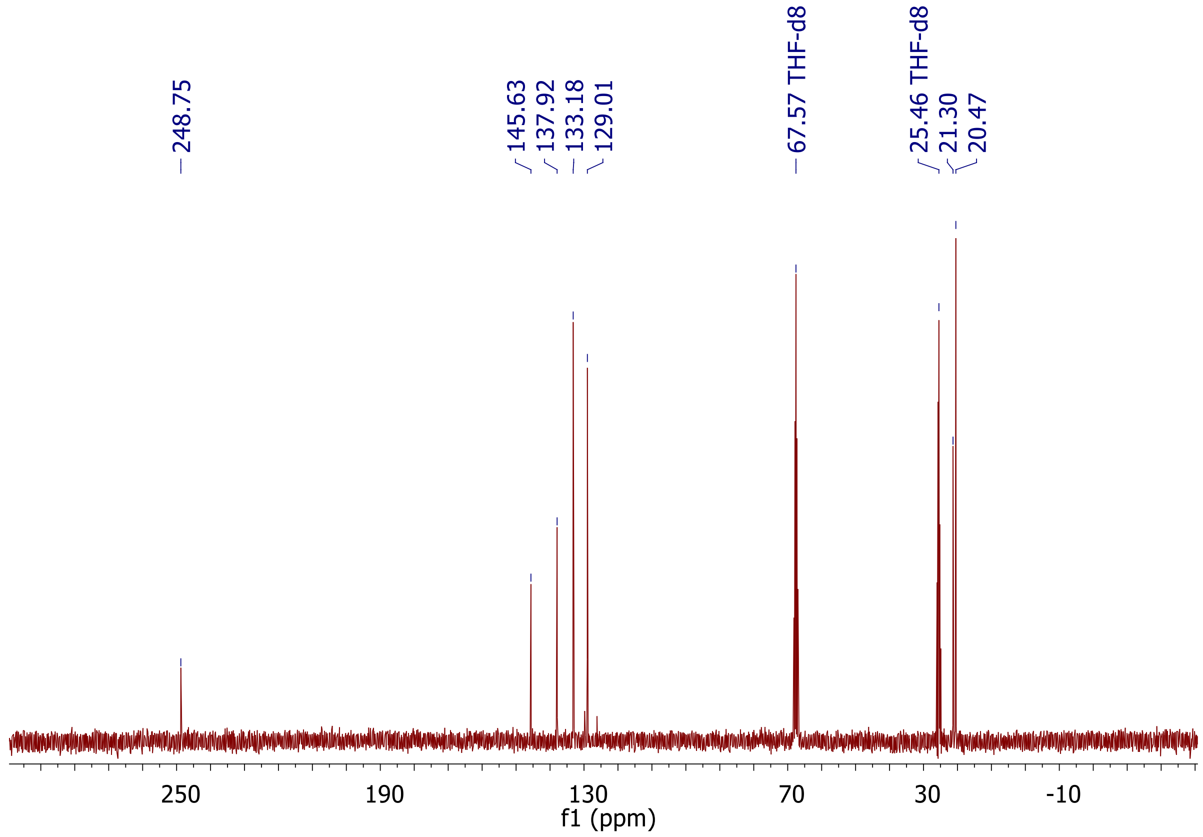

Figure S9: ^{1}H- and ^{13}C-NMR spectra of **7** (C_6D_6 solution, vs ext. TMS, ppm)

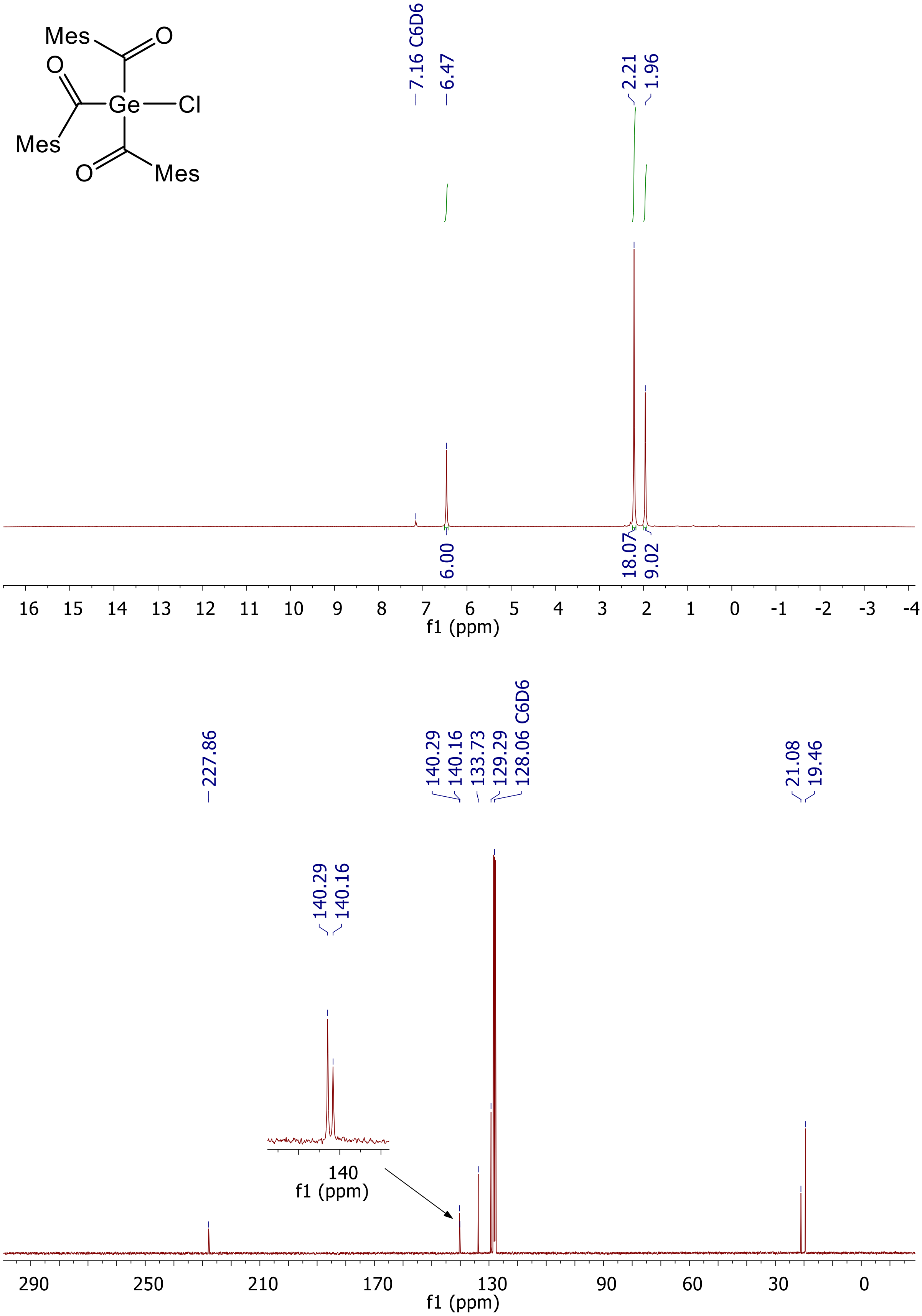

Figure S10: ^{1}H- and ^{13}C-NMR spectra of **8** (C_6D_6 solution, vs ext. TMS, ppm)

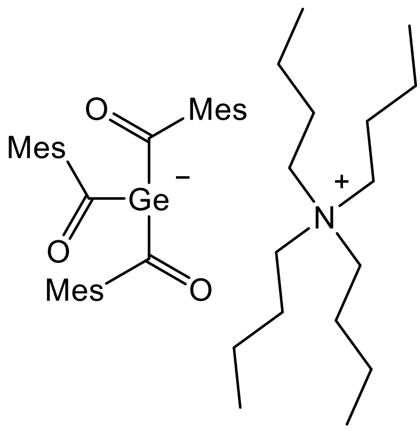

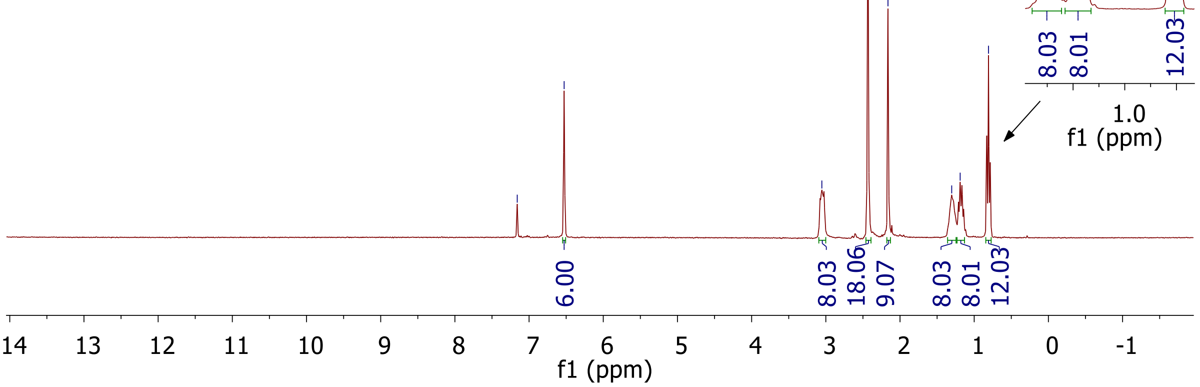

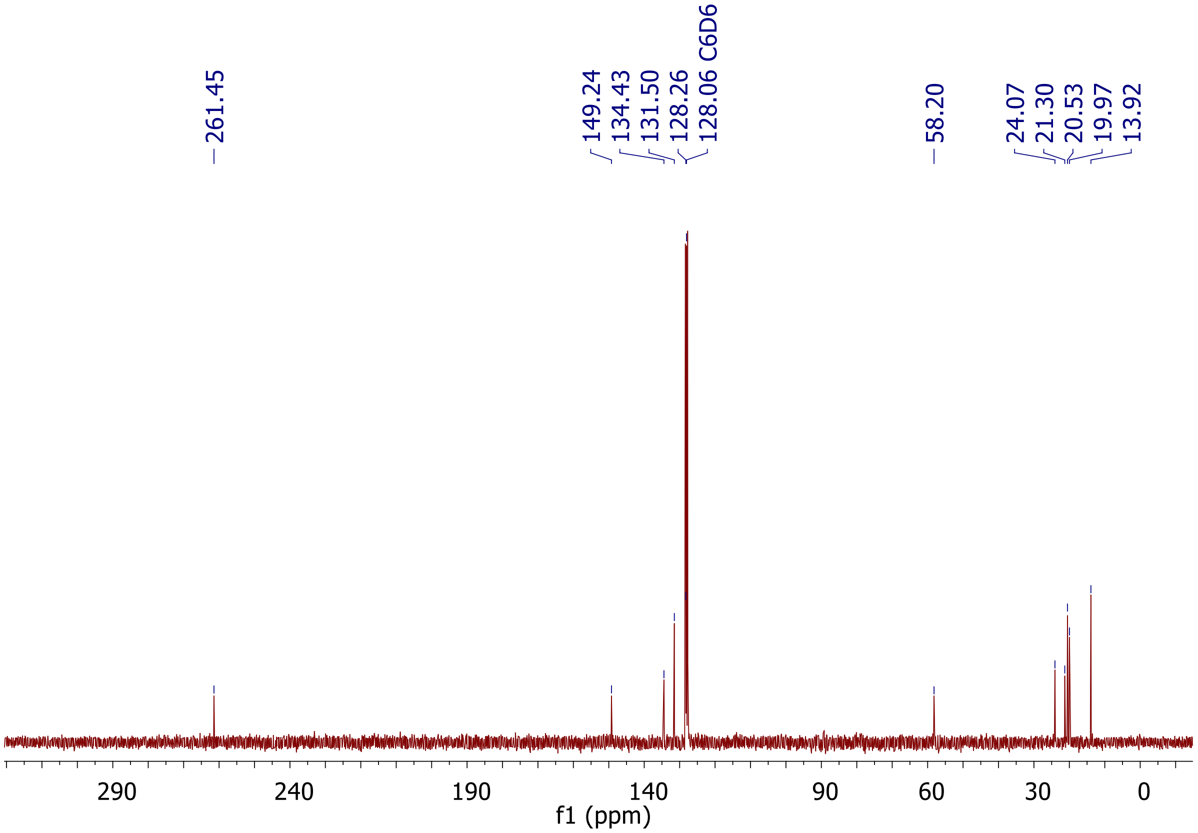

Figure S11: ^{1}H- and ^{13}C-NMR spectra of **9** (C_6D_6 solution, vs ext. TMS, ppm)

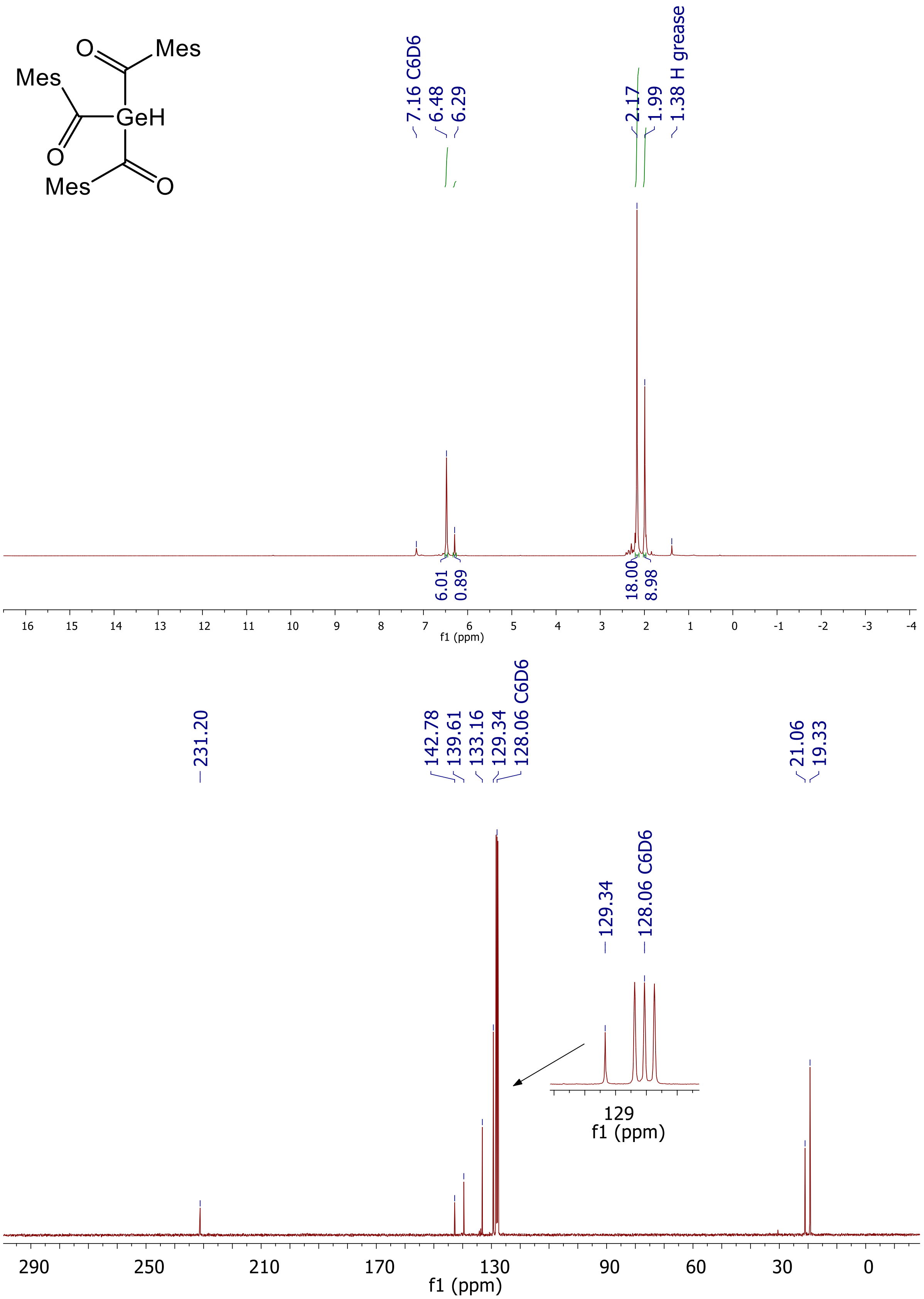

Figure S12: ^{1}H-NMR spectra of **10** (C_6D_6 solution, vs ext. TMS, ppm)

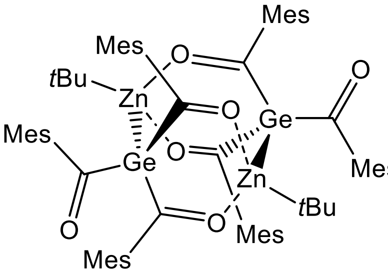

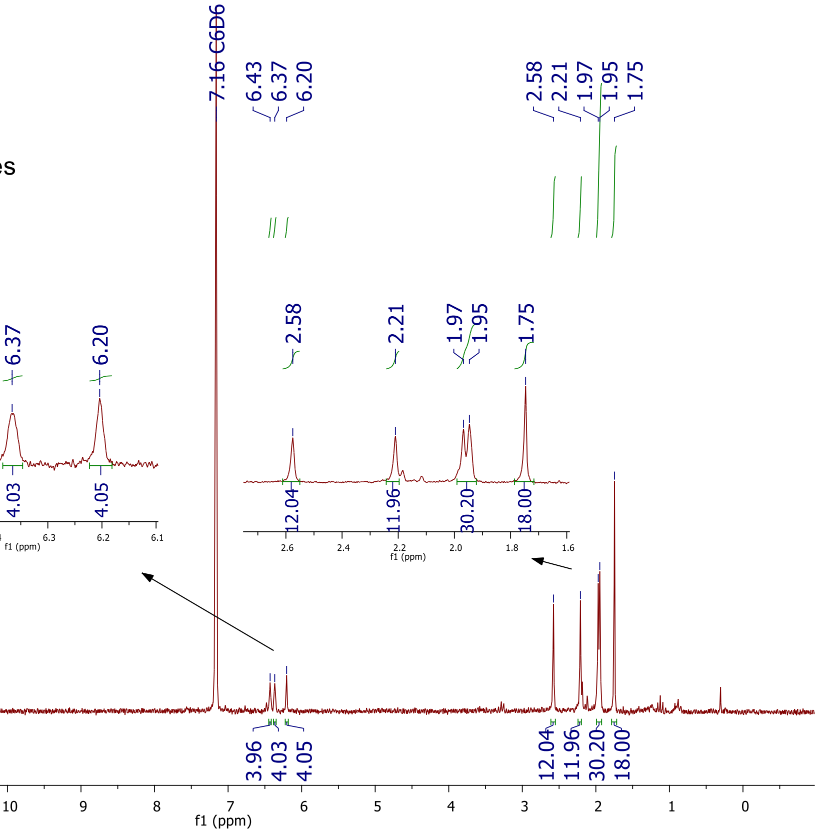

Figure S13: ^{1}H- and ^{13}C-NMR spectra of **11** (C_6D_6 solution, vs ext. TMS, ppm)

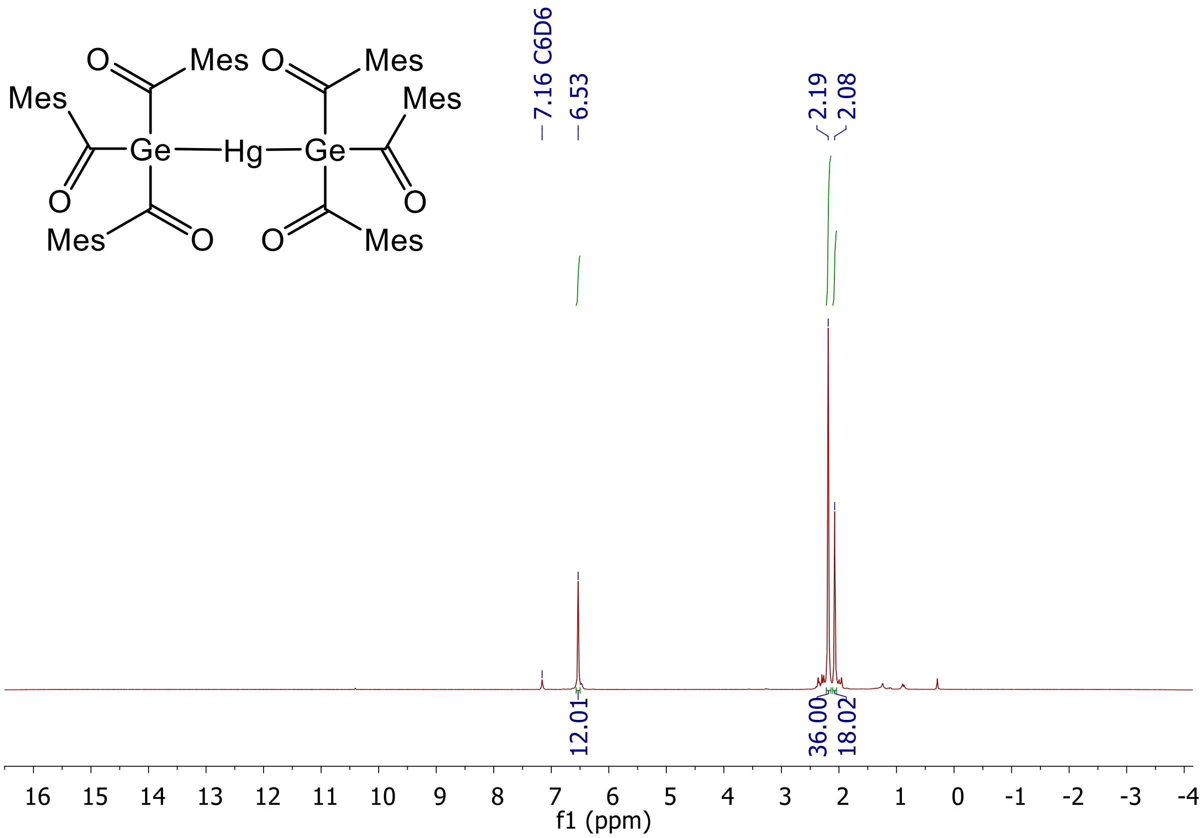

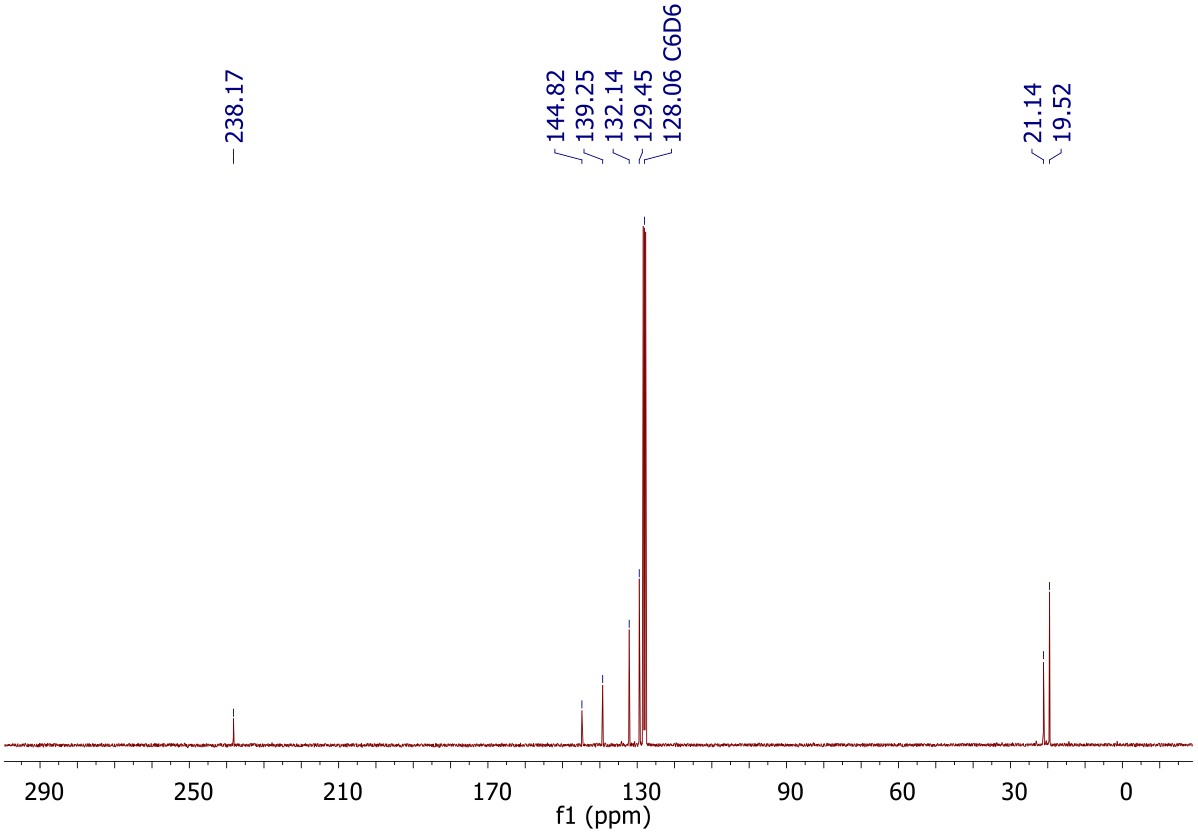

UV-vis Spectroscopy

Figure S14: UV-vis Spectrum of **5** (c = 5 x 10^{-5} M) measured in THF.

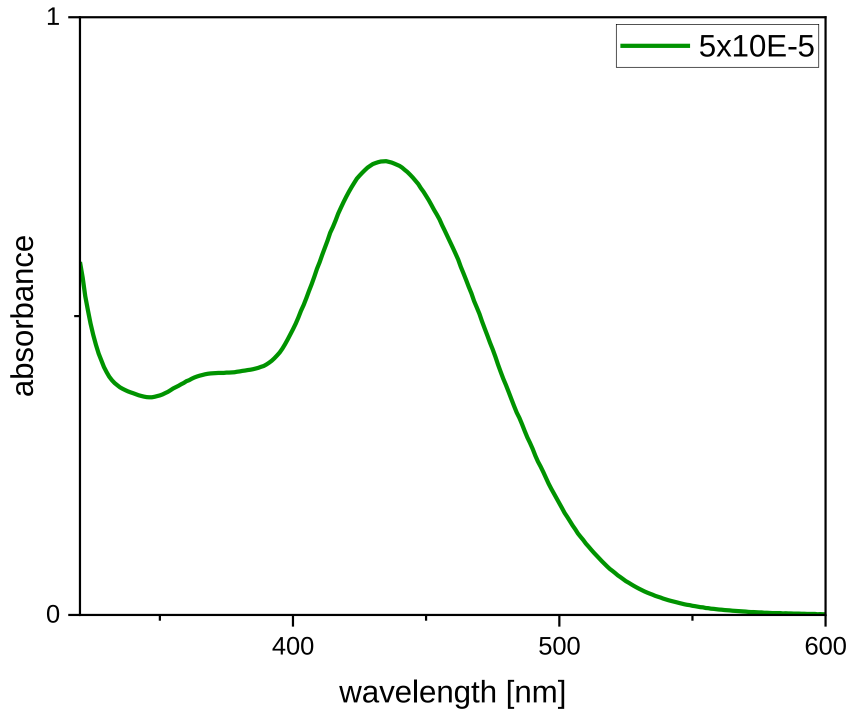

Figure S15: UV-vis Spectrum of **6** (c = 5 x 10^{-4} M) measured in THF.

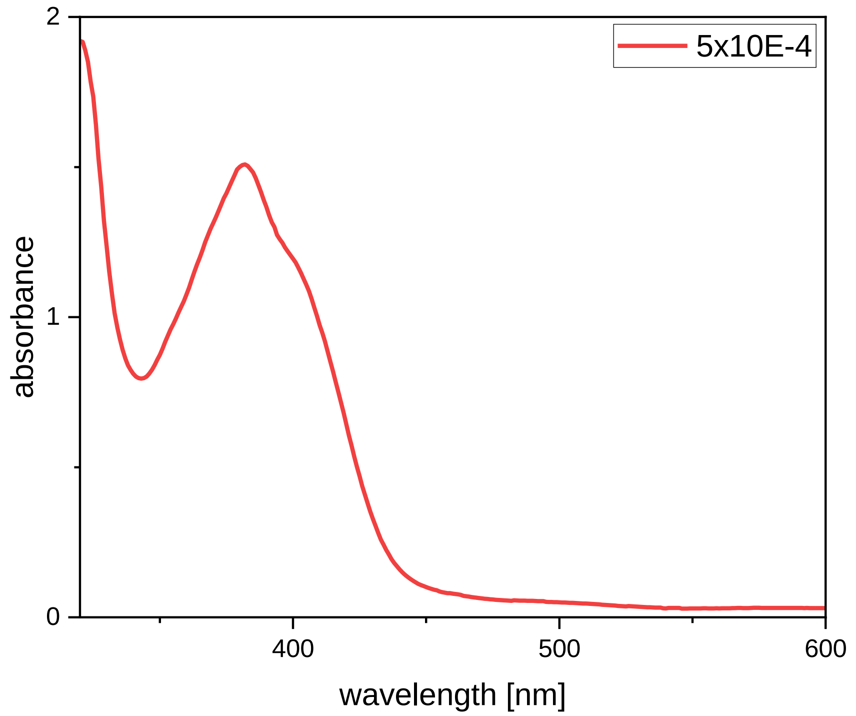

Figure S16: UV-vis Spectrum of **7** (c = 5 x 10^{-4} M) measured in THF.

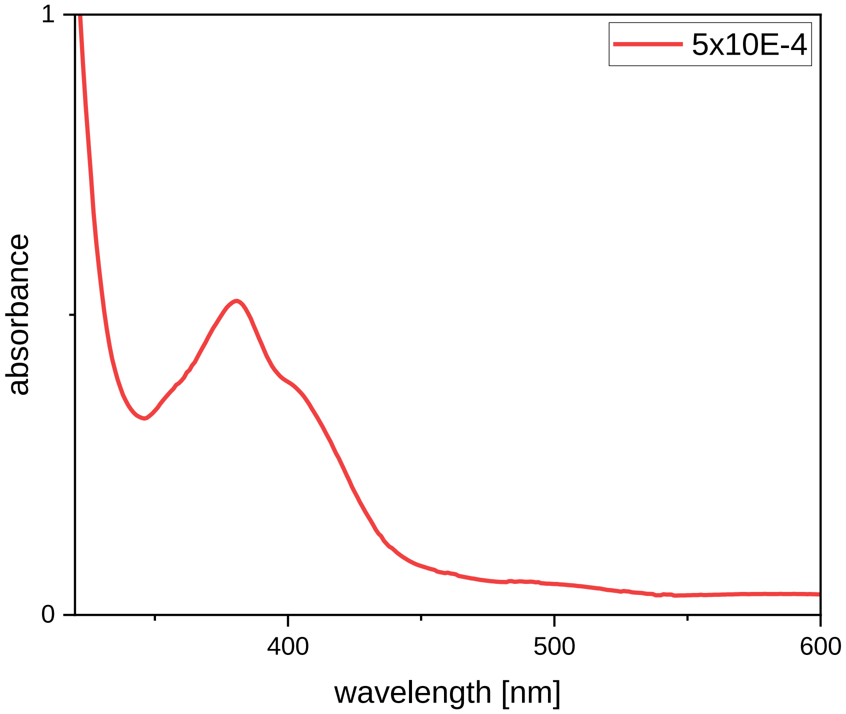

Figure S17: UV-vis Spectrum of **8** (c = 1 x 10^{-4} M) measured in THF.

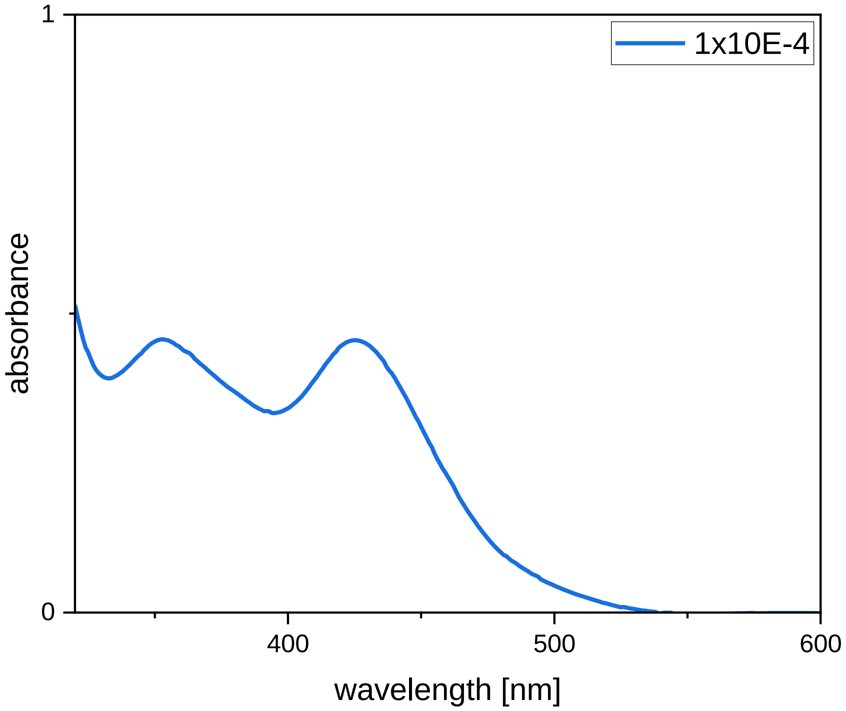

Figure S18: UV-vis Spectrum of **9** (c = 5 x 10^{-4} M) measured in toluene.

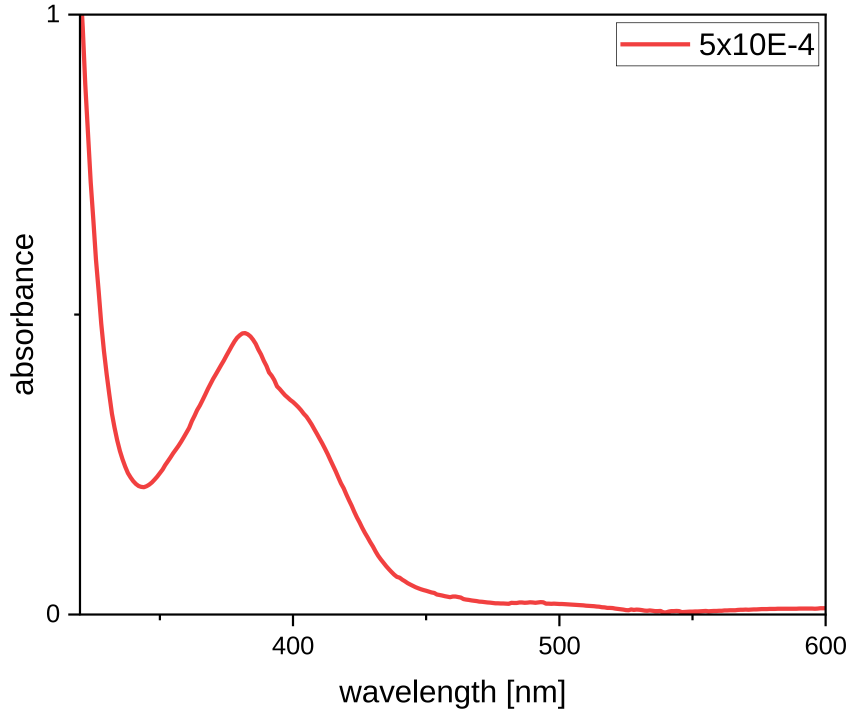

Figure S19: UV-vis Spectrum of **11** (c = 1 x 10^{-4} M) measured in *n*-hexane.

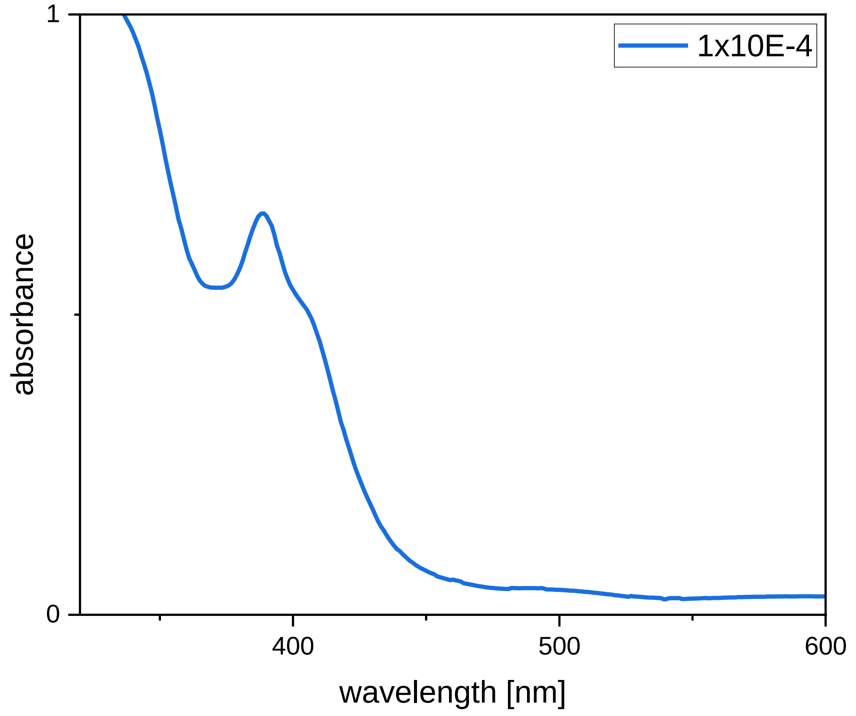

X-ray Crystallography

Table S1. Crystallographic data and details of measurements for compounds **5**, **6**, **7** and **10**.

Compound	2174956 (5)	2174957 (6)	2174958 (7)	2174959 (10)
**Formula**	$C_{76}H_{98}Ge_2MgO_{10}$	$C_{76}H_{98}Cl_4Ge_2K_{1.97}O_{10}Zn_2$	$C_{30}H_{33}ClGeO_3$	$C_{43}H_{51}GeO_3Zn$
**Fw (g mol^{-1})**	1341.03	1667.46	549.60	753.79
a **(Å)**	12.5590(5)	31.390(3)	24.5964(13)	12.0378(7)
b **(Å)**	22.0195(9)	12.6678(12)	14.5403(7)	13.6631(8)
c **(Å)**	25.5617(10)	19.8054(18)	28.0803(15)	13.9423(8)
α **(°)**	90	90	90	62.053(2)
β **(°)**	91.557(2)	91.220(5)	107.692(3)	81.345(3)
γ **(°)**	90	90	90	72.546(3)
V **(Å^3)**	7066.3(5)	7873.8(13)	9567.6(9)	1932.3(2)
Z	4	4	14	2
**Crystal size (mm)**	0.19 × 0.14 × 0.1	0.25 × 0.22 × 0.19	0.18 × 0.15 × 0.110	0.18 × 0.15 × 0.11
**Crystal habit**	Block, yellow	Block, yellow	Block, yellow	Block, yellow
**Crystal system**	monoclinic	monoclinic	monoclinic	triclinic
**Space group**	$P2_1/n$	C2/c	$P2_1$	P-1
**d_{calc} (Mg m^{-3})**	1.261	1.407	1.335	1.296
**μ (mm^{-1})**	0.916	1.654	1.247	1.435
T **(K)**	99.99	100 (2)	99.98	99.98
**2θ range (°)**	4.026 to 53.998	4.818 to 58.352	1.522 to 53.998	4.278 to 58.086
***F*(000)**	2840.0	3456.0	4004.0	790
**R_{int}**	0.0806	0.1140	0.0600	0.0666
**No. of measured and independent [*I* > 2s(*I*)] reflections**	214087, 15401	76991, 10538	330669, 41712	49781, 10198
**No. of parameters, restraints**	1220, 635	443, 0	2290, 631	558,58
**Δ›$_{max}$, Δ›$_{min}$ (e Å^{-3})**	1.18/-0.73	0.55/-0.49	1.32/-2.43	0.46/-0.79
**R1, wR2 (all data)**	R1 = 0.0750<br>wR2 = 0.1228	R1 = 0.0876<br>wR2 = 0.0913	R1 = 0.0841<br>wR2 = 0.1753	R1 = 0.0625<br>wR2 = 0.0732
**R1, wR2 (>2σ)**	R1 = 0.0549<br>wR2 = 0.1140	R1 = 0.0444<br>wR2 = 0.0795	R1 = 0.0760<br>wR2 =0.1714	R1 = 0.0353<br>wR2 =0.0662
